# *Galleria mellonella*–intracellular bacteria pathogen infection models: the ins and outs

**DOI:** 10.1093/femsre/fuad011

**Published:** 2023-03-11

**Authors:** Masanori Asai, Yanwen Li, Sandra M Newton, Brian D Robertson, Paul R Langford

**Affiliations:** Section of Paediatric Infectious Disease, Department of Infectious Disease, St Mary’s campus, Imperial College London, London W2 1PG, United Kingdom; Section of Paediatric Infectious Disease, Department of Infectious Disease, St Mary’s campus, Imperial College London, London W2 1PG, United Kingdom; Section of Paediatric Infectious Disease, Department of Infectious Disease, St Mary’s campus, Imperial College London, London W2 1PG, United Kingdom; Centre for Bacterial Resistance Biology, Department of Infectious Disease, South Kensington campus, Imperial College London, London SW7 2AZ, United Kingdom; Section of Paediatric Infectious Disease, Department of Infectious Disease, St Mary’s campus, Imperial College London, London W2 1PG, United Kingdom

**Keywords:** *Galleria mellonella*, intracellular pathogen, mycobacteria, virulence, antimicrobial, insect

## Abstract

*Galleria mellonella* (greater wax moth) larvae are used widely as surrogate infectious disease models, due to ease of use and the presence of an innate immune system functionally similar to that of vertebrates. Here, we review *G. mellonella–*human intracellular bacteria pathogen infection models from the genera *Burkholderia, Coxiella, Francisella, Listeria*, and *Mycobacterium*. For all genera, *G. mellonella* use has increased understanding of host–bacterial interactive biology, particularly through studies comparing the virulence of closely related species and/or wild-type versus mutant pairs. In many cases, virulence in *G. mellonella* mirrors that found in mammalian infection models, although it is unclear whether the pathogenic mechanisms are the same. The use of *G. mellonella* larvae has speeded up *in vivo* efficacy and toxicity testing of novel antimicrobials to treat infections caused by intracellular bacteria: an area that will expand since the FDA no longer requires animal testing for licensure. Further use of *G. mellonella*–intracellular bacteria infection models will be driven by advances in *G. mellonella* genetics, imaging, metabolomics, proteomics, and transcriptomic methodologies, alongside the development and accessibility of reagents to quantify immune markers, all of which will be underpinned by a fully annotated genome.

## Teaser

Infection of larvae of the insect *Galleria mellonella* (greater wax moth) with bacteria that reside inside human cells and cause disease (intracellular pathogens) is valuable in screening for new antibiotics and identifying bacterial factors necessary to cause disease.

## Introduction

In recent years, *Galleria mellonella* (greater wax moth) larvae have been used extensively as a surrogate model for infection and to assess antimicrobial activity (Tsai et al. 2016, Cutuli et al. 2019, Piatek et al. 2020, Dinh et al. 2021), in part driven by a movement towards reduction, replacement, and refinement (3Rs) of the use of vertebrate animals in scientific experimentation (Graham and Prescott 2015). While there are natural pathogens of *G. mellonella*, such as the fungus *Beauveria bassiana* (Vertyporokh et al. 2020), the most recent work involving *G. mellonella* infection has been with non-native (human) pathogens. It was estimated that *G. mellonella* larvae were used as an infection model with 65+ strains of bacteria and fungi combined, and there has been a rapid increase in research papers in the last decade (Dinh et al. 2021). Here, our aim is to review studies that have investigated the interaction of *G. mellonella* with mycobacteria and other examples of intracellular bacterial pathogens, predominantly those that infect man, either as an infection model and/or in the evaluation of virulence factors and/or antimicrobial agents. Intracellular bacterial pathogens require host cells for replication and proliferation as part of the infectious process (Eisenreich et al. 2019). The main emphasis will be on *Mycobacterium* spp., but selected other pathogens including, *Coxiella burnetii*, and from the genera, *Burkholderia, Francisella*, and *Listeria* are also considered. An excellent review of *G. mellonella*–*Legionella pneumophila* interaction has recently been published (Frankel and Schroeder 2019) and is not considered here. The structure of this review is to give a brief introduction to *G. mellonella* and the reasons that it is widely used as a surrogate model in infectious disease research, followed by, for each bacterial genus or species, a brief introduction to their importance and a review of the studies describing their interaction with *G. mellonella*. Finally, we compare the work types undertaken with the pathogens of interest and discuss research gaps and future prospects.

## Galleria mellonella

The destructive properties of *G. mellonella* to honeybee colonies have long been recognized: they were described by Aristotle in *c*. 350 bc in the ‘History of Animals’ as a moth that ‘is never stung by a bee and can only be got out of a hive by fumigation’ and engenders a caterpillar nicknamed the ‘borer’ (Aristotle 350bc). Taxonomically, the greater wax moth is classified as a member of the order Lepidoptera, family Pyralidae, and subfamily Galleriinae. *Galleria mellonella* has been found on all continents except Antarctica. Precise figures are difficult to estimate, but the insect is considered to cause a significant worldwide economic cost, especially in Africa and Asia (Kwadha et al. 2017). *Galleria mellonella* is a holometabolous insect, i.e. its life cycle has four developmental stages: egg, larva, pupa, and adult. Eggs are laid typically in batches of 50–150, and soon after hatching, larvae move from the cracks and crevices of the beehive to feed on honeycomb, which contains a significant amount of beeswax, some honey, bee larvae exuviae (moulted exoskeleton), and pollen residues (Kwadha et al. 2017, Wojda 2017). Protection of the feeding *G. mellonella* larvae from bees is afforded by the spinning of silken tubes, and the net result is honeycomb destruction (Wojda et al. 2020), which can occur within 7 days of colonization (Hosamani et al. 2017). Typically, there are seven (L1–L7) larval instars over an ∼45-day period (Wojda et al. 2020), with the last two exhibiting the most intensive growth (Ellis et al. 2013). The last instar larvae move to safe places, e.g. the outer surface of bee frames, where they spin a silken pupal cocoon which protects against worker bees and provides an environment for pupal development and eventually the emergence of adults (Wojda et al. 2020). Once adults appear, and after mating, egg laying in the dark cracks and crevices of the beehive (Charriere and Imdorf 1999) occurs soon after (Nielsen and Brister 1977). Females lay their eggs at night. Adults cannot consume food as their mouthparts are degenerate, and thus they live for a short time, 7–30 days (Hosamani et al. 2017). *Galleria mellonella* larvae were found to rapidly biodegrade polyethylene to ethylene glycol via a phenol oxidase present in saliva, which is the basis for a method to decrease the environmental impact of the trillions of plastic bags produced each year (Sanluis-Verdes et al. 2022).

**Figure 1.**
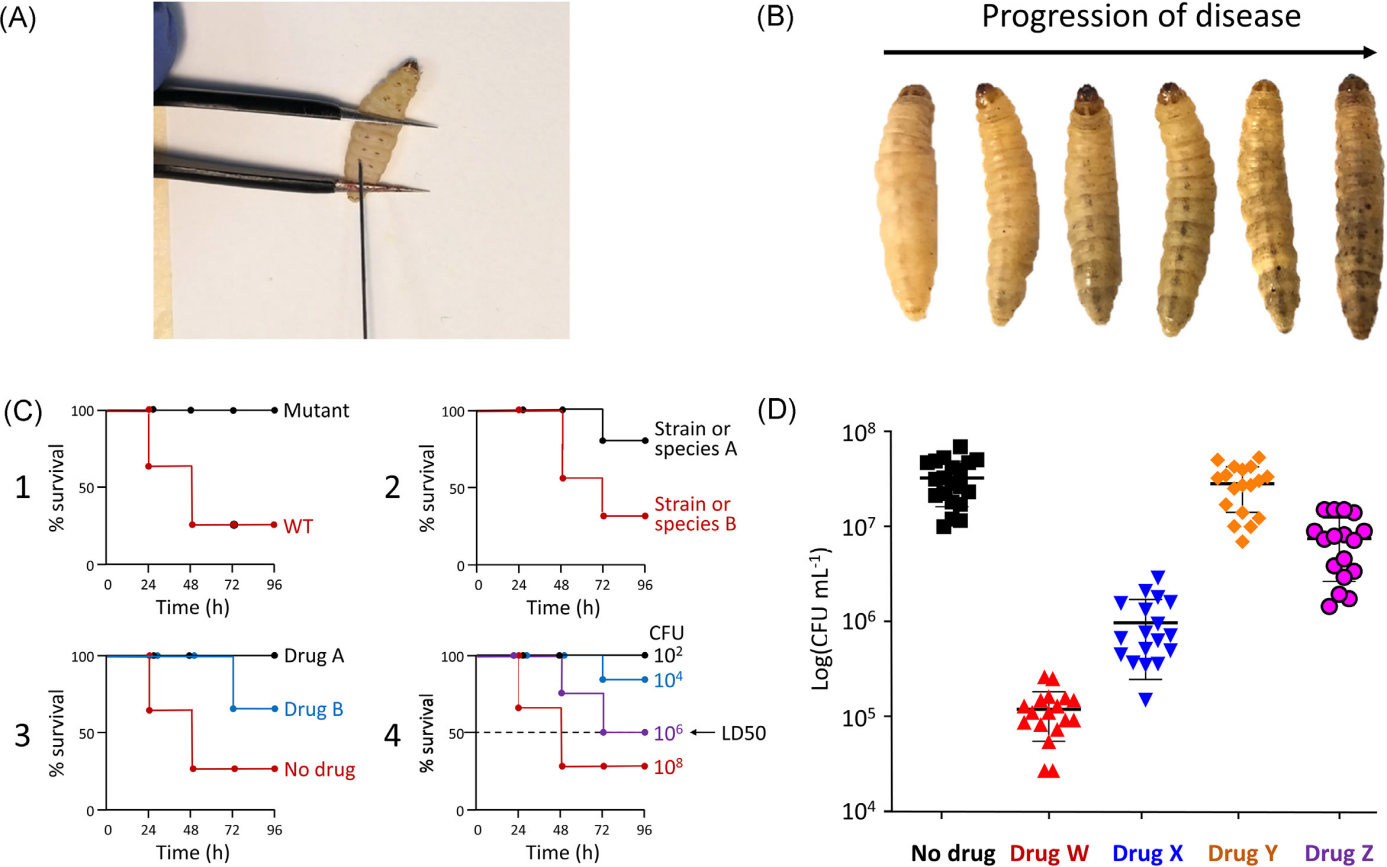
From infection to treatment – *G. mellonella* data acquisition. (A) *Galleria mellonella* larvae are infected/treated via injection using a microsyringe for accurate dosing. (B) Larval melanization (darkening of the cuticle) can quantitatively monitor disease progression. (C) The most common readouts found in *G. mellonella*-based infection studies are Kaplan–Meier (KM) survival plots of percentage survival versus time, formulated originally by and named after Kaplan and Meier (1958). C1-4 shows examples. (C1) Comparative virulence of wild-type (WT) versus an isogenic mutant with an equivalent dose. (C2) Comparative virulence of strain or species A versus strain or species B at an equivalent dose. (C3) Drug efficacy. Typically, *G. mellonella* larvae are infected, and at the appropriate time, drugs are injected, and their ability to prevent lethal infection is determined. Different doses may be used. In some studies, drugs are given before or immediately after infection. In the example shown, Drug A is more efficacious than Drug B. Typically, viable counts are determined to validate this assertion. (C4) LD50, i.e. the inoculum size that kills 50% of larvae may also be determined. In the example shown, the LD50 is 10^6^ CFU at 96 h. A variation is the LT50, i.e. the time to kill 50% of larvae. All methods described above are routine for the analysis of interactions of *G. mellonella* with intracellular bacterial pathogens included in this review. In the case of (C1–C3), a quantitative method to determine viability, e.g. CFU or luminescence, is also typically used, and a drug treatment example is shown (D). Viability may be determined at all or just the last time point. Depending on the study aims, proteomic (e.g. liquid chromatography-tandem mass spectrometry, SDS-PAGE) and/or gene expression (e.g. transcriptomic, RT-qPCR), histological, and microscopy analyses of *G. mellonella* and/or the intracellular bacterium of interest, may also be done.

In recent decades the use of *G. mellonella* larvae as a surrogate model for infection and to assess antimicrobial activity reflects the many recognized advantages, including (1) low cost to acquire and maintain, (2) no requirement for specialized equipment and facilities, (3) incubation at 37°C, i.e. the optimum temperature of many human pathogens, (4) lack of ethical constraints in their use, (5) a rapid infection cycle allowing for mid to high throughput data generation, and (6) possess only an innate immune system, which can be studied without any influence from adaptive immunity (Asai et al. 2019a). The use of nonmammalian hosts allows faster early-stage results where ‘speed is king and a fail-fast-fail-safe mantra rules’ (Hunter 2023). Compared to other invertebrate hosts such as the nematode *Caenorhabditis elegans* and *Drosophila melanogaster* (fruit fly) used in infectious disease research (reviewed in Glavis-Bloom et al. 2012), including with intracellular bacterial pathogens, points (2) and (3) are particularly advantageous when working with *G. mellonella*. Figure 1 shows a diagrammatic representation of basic methods used in *G. mellonella*–pathogen infection studies, including those with intracellular bacteria, and examples of Kaplan–Meier survival curves – the most common readout. The innate immune system shares functional and anatomical similarities to those found in man (Wojda 2017) and comprises cellular and humoral compartments (Hillyer 2016). Haemocytes (analogous to mammalian white blood cells), of which five types have been identified and whose functions include phagocytosis, nodulation, and encapsulation, are the drivers of the cellular response (Pereira et al. 2018). Activation of the humoral response occurs when pathogen/damage-associated molecular patterns, such as lipopolysaccharide, are recognized by host pattern recognition receptors (e.g. Toll-like receptors), which in turn induce signalling cascades (Browne et al. 2013). Such interactions lead to the expression of antimicrobial peptides (AMPs) (reviewed in Mikulak et al. 2018, Andrejko et al. 2021) and secretion of reactive oxygen/nitrogen species and hydrogen peroxide into the haemolymph found in the haemocoel (analogous to blood/blood vessels). In addition, activation of the prophenol oxidase (PPO) cascade can result in melanization, which controls and localizes infection through melanin deposition and production of phenolic compounds, which in function is considered similar to the mammalian complement cascade (Browne et al. 2013). Finally, colonization resistance, i.e. the ability of the microbial flora to prevent infection by other microorganisms, e.g. through the production of bacteriocins or via niche exclusion, also contributes to immunity (Dinh et al. 2021).

## *Galleria mellonella–*mycobacteria infection models

### Introduction to mycobacteria and the need for new infection models

**Figure 2.**
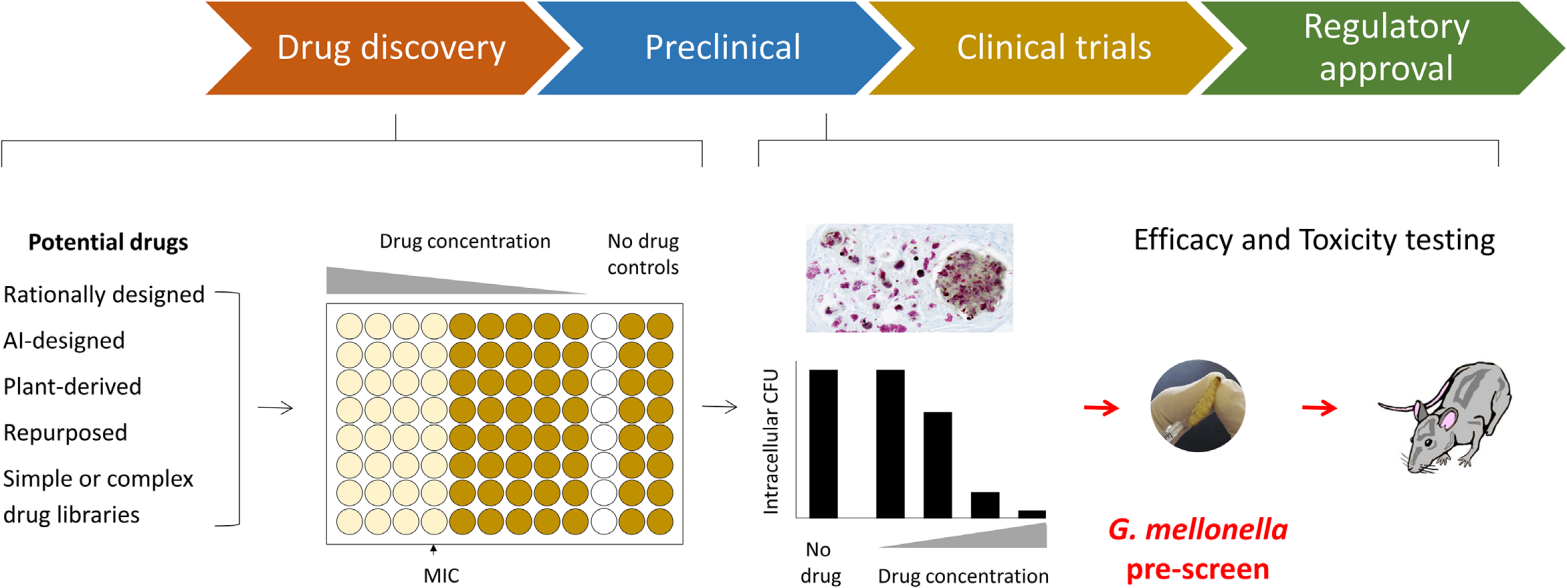
Overview of the *G. mellonella-*enhanced mycobacterial drug development pipeline. The chevron arrows at the top show the overall drug development pipeline. During the drug discovery phase, different routes identify potential drugs and their minimum inhibitory concentration (MIC) determined against mycobacteria in low or high throughput screens *in vitro*. The ability of promising compounds to kill mycobacteria growing intracellularly, e.g. in mouse J774 or RAW 264.7 cell lines, is then tested. In a conventional pipeline, efficacious compounds are then tested for their ability to cure *M. tuberculosis*-infected mice. Dependent on the study design, the preclinical stage may involve seeking basic toxicity data. The *G. mellonella*-enhanced pipeline involves a prescreen (in red) following intracellular efficacy testing, where the efficacy and toxicity of promising compounds are tested in *M. tuberculosis*-infected and noninfected larvae, respectively. Compounds that are efficacious and nontoxic in *G. mellonella* would then typically proceed to testing in mice. However, a recent FDA decision means licensure no longer requires animal data. The *G. mellonella*-enhanced pipeline enables promising efficacious nontoxic compounds to be identified at an earlier stage, shortening the overall drug development process, which results in a reduction of mammals in research and associated costs. AI = artificial intelligence.

**Figure 3.**
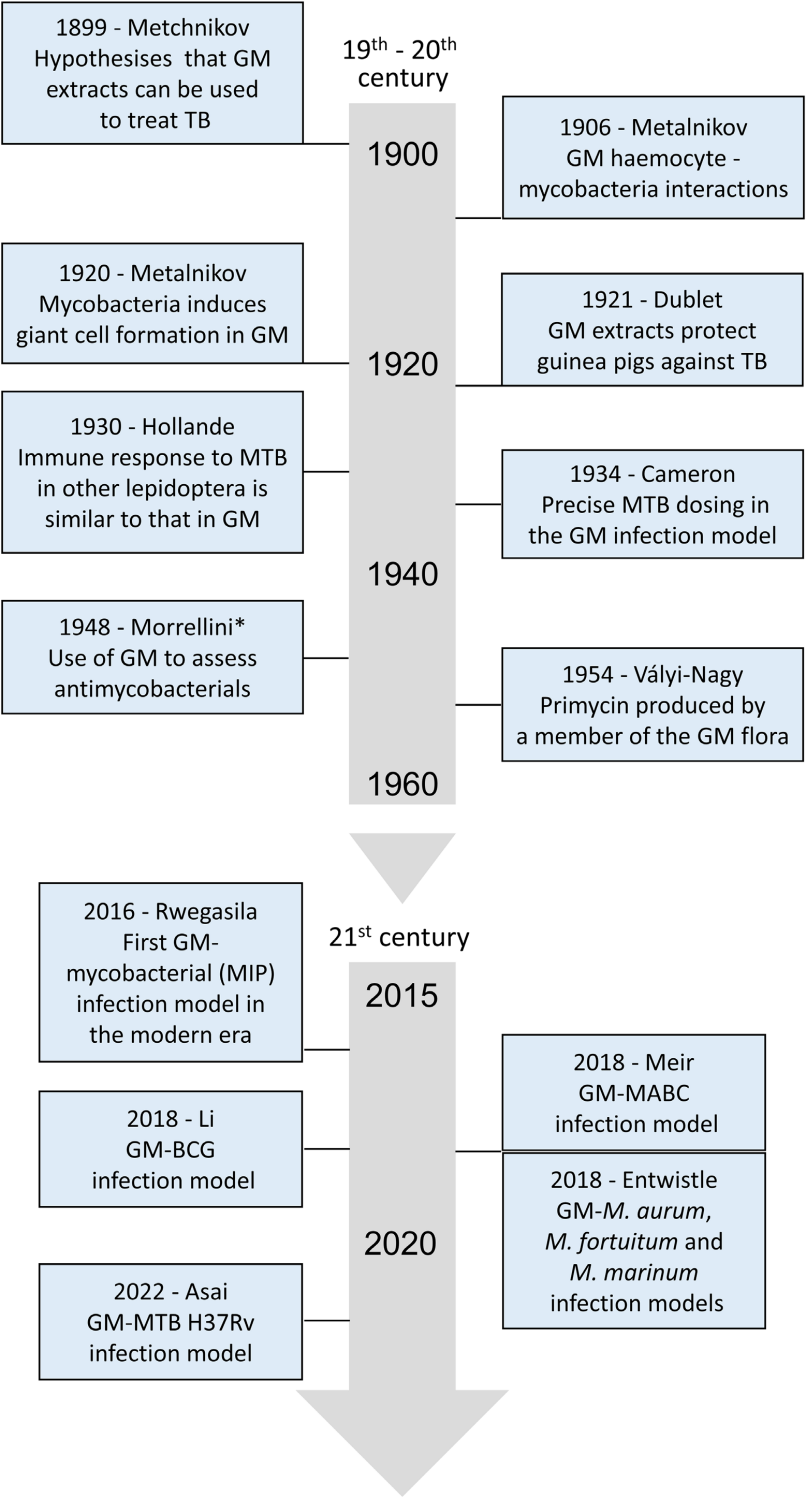
Timeline of important historical advances in *G. mellonella*–mycobacterial research in the 19th and 20th centuries (top) and 21st century (bottom). Timelines not to scale. Each box lists the year, the first author of the associated paper, and the advance. See the main text for full details of each advance. *Experiments described in the Ferrari and Barbaro (1964) review of the work of Morrellini and Cattaneo. BCG = bacille Calmette–Guérin; GM = *G. mellonella*; MABC = *M. abscessus* complex; MIP = *M. indicus pranii*; MTB = *M. tuberculosis*; and TB = tuberculosis.

The mycobacteria are a large genus of around 200 organisms within the family Actinobacteria. While the vast majority are saprophytes living in the environment, a small minority are infamous as agents of diseases recognized throughout human history. These include *Mycobacterium leprae*, the cause of leprosy, known by its skin lesions and deformities and feared since ancient times, and *Mycobacterium tuberculosis*, the cause of tuberculosis, but known by many names including consumption, phthisis, the white death, the graveyard cough, the King’s Evil, and the ‘Captain of all these men of Death’. Some are also animal pathogens, e.g. *Mycobacterium avium, Mycobacterium bovis* [part of the *M. tuberculosis* Complex, (MTBC)], and *Mycobacterium marinum* that infect birds, cows, and fish, respectively. There are also nontuberculous mycobacteria (NTM) associated with human pulmonary diseases – see individual sections for further details. Treatment of all human mycobacterial infections is complex, requiring multiple antimicrobials for extended periods – the current short-course therapy for tuberculosis treatment is 6 months, but considerably longer for treatment of antimicrobial-resistant infections (Dartois and Rubin 2022). Major efforts are underway to find shorter drug regimens to treat sensitive and resistant infections and to develop vaccines that work in the highest-risk populations. Current WHO targets of the End TB strategy are ambitious, i.e. by 2035, to reduce the incidence rate by 90% compared to 2015 and have no families facing catastrophic losses due to the disease (Stop TB Partnership 2022). However, reaching these targets will not only require the discovery, development and rapid uptake of new tools, interventions, and strategies, but also research to optimize implementation, impact, and promote innovation. Preclinical studies of new drugs and vaccines have been hampered by the available animal models, which do not adequately reflect key aspects of human disease (Singh and Gupta 2018). Consequently, there is a need for faster, low-cost, more readily available models to evaluate new interventions at an earlier stage before embarking on costly and involved complex animal models. Larvae of the greater wax moth *G. mellonella* may be an ideal model to fill this gap (Fig. 2). Figure 3 shows a timeline of advances in *G. mellonella*–mycobacteria research, with further details in the sections below.

### Pioneering studies of *G. mellonella*–mycobacteria interactions in the 19th and 20th centuries

The first entry of ‘*Galleria mellonella*’ in PubMed is in 1938 (Smith 1938), which describes the use of X-rays to induce mutations in traits such as body and eye colour, and for ‘*Galleria mellonella Mycobacterium*’ in 1952 (Terni and Gargani 1952), which describes the survival of *Mycobacterium lepraemurium* (which causes leprosy in mice) in *G. mellonella* larvae. However, before 1938, considerable literature exists not indexed in PubMed describing work with *G. mellonella* and mycobacteria, which have received comparatively little attention. An early pioneer was Ilya Metchnikov, the recipient (jointly with Paul Ehrlich) of the Nobel Prize in Physiology or Medicine in 1908 in recognition of his work on the role of phagocytosis of microbes in host defence, and who is considered the ‘Father of Natural Immunity’ (Gordon 2008). Metchnikov was aware of Robert Koch’s work on the discovery and pathogenesis of *M. tuberculosis*, having met him many times (Heifets 1982), and had extensively worked with insects in his work on phagocytosis. Metchnikov knew that *M. tuberculosis* grown on agar plates has a waxy consistency and that *G. mellonella* can break down beeswax in hives to survive. He reasoned that the *G. mellonella* enzymes that break down beeswax might also act on *M. tuberculosis* and be used as a treatment for tuberculosis (Metchnikov 1899). This line of reasoning started a new area of research, which ultimately led to commercial products that are still used today, albeit with unproven and proven efficacy for tuberculosis and bacterial skin infections, respectively (see section *G. mellonella* derived products for the treatment of tuberculosis).

Significant advances in our understanding of *G. mellonella*–mycobacteria interactions arose from Serguei Metalnikov’s work at the Institute Pasteur from 1919 onwards, where he led the insect immunity group (reviewed in Carton 2019). He had previously written an extensive monograph on *G. mellonella* (Metalnikov 1908) that included descriptive analyses of anatomy and physiology, the structure of the digestive organs, nutrition, experiments with injections of dyes, and phagocytosis including reference to, but no detail of, experiments with mycobacteria. He knew of Metchnikov’s work via an internship 1906 with him (Carton 2019). Metalnikov (also occasionally referred to as Metalnikoff or Metalnikow) briefly mentioned *G. mellonella*–mycobacteria interactions in 1906, describing the bacteriolytic properties of *G. mellonella* haemolymph for tubercle bacilli and that older cultures are more sensitive and wondered ‘which is the substance’ that has the bacteriolytic activity. In addition, he found that *G. mellonella* infected with the fish pathogen *M. marinum* died when incubated at 15–20°C but not at 37°C, a result that he attributed to the bacterium either growing poorly or not at all at the higher temperature (Metalnikoff 1906, Metalnikov 1911).

Arguably, his most influential paper analysed the effect of low and high doses of 35 pathogenic or saprophytic bacteria of humans or mammals on the survival of *G. mellonella* (Metalnikov 1920). Microbes were classified into three categories [Group A contained those with complete immunity (resistant to low and high doses); Group B those with incomplete immunity (susceptible to high doses); and Group C those with no immunity (susceptible to low and high doses)]. Group A contained: B. tuberculeux humain ( = *M. tuberculosis*), B. tuberculeux bovin ( = *M. bovis)*, B. tuberculeux aviaire ( = *M. avium*), and B. tuberculeur pisciaire ( = *M. marinum*) (Metalnikov 1920). Initial follow-up studies focussed on mycobacteria, primarily the ‘tubercle bacillus’ ( = *M. tuberculosis*) because ‘it is the least virulent for the caterpillar’ and ‘…in immunity to tuberculosis that the caterpillar reveals all of its antimicrobial defences’, and he posed the question ‘But what is the cause of this extraordinary immunity?’ Time course analyses established that all the mycobacterial species listed had a similar response comprising initial phagocytosis by *G. mellonella* leukocytes ( = haemocytes), followed by the formation of giant cells (called plasmodes) and capsules surrounding bacilli, which were associated with mycobacterial killing. He considered giant cell and capsule formation analogous to granulomas that form in humans in response to *M. tuberculosis*. Such structures formed in larvae, chrysalids, and adult moths (Metalnikow 1925). In addition, Metalnikov noted that ‘all capsules contain a brown–black pigment’ (Metalnikov 1920), most likely melanin, although Hollande pointed out that this was not unique to the Koch bacillus (Hollande 1920). A similar conclusion, based on experiments with mycobacterial cow, dog, and human isolates, i.e. ‘Koch’s bacillus actually behaves as a simple foreign body’, was made by Redaelli (1929). Other *G. mellonella*–mycobacteria challenge experiments were carried out by Feissinger (1920), Redaelli (1929), Hollande (1930), and Cameron (1934). Feissinger confirmed Metalnikov’s observations, describing phagocytosis of Koch’s bacillus (*M. tuberculosis*) in the haemolymph of *G. mellonella* after 30 min, and the presence of brownish vacuoles containing bacteria within 3 h (Feissinger 1920). Redaelli (1929) investigated the interaction of a strain of *M. tuberculosis* and two of *M. bovis*, one from a cow and one from a dog, with *G. mellonella*. Rapid phagocytosis and killing of the bacteria in haemocytes occurred. Hollande carried out challenge studies with Koch’s bacillus, *M. avium, M. bovis, M. marinum*, and nonpathogenic fast-growing *Mycobacterium smegmatis*, with most of his studies with *M. avium* due to its ease of handling and fast growth (Hollande 1930). Hollande also injected Koch’s bacillus into larvae of other moths and butterflies including *Aporia crataegi* (black-veined white butterfly), *Lithosia complana* (scarce footman moth), *Pieris brassicae* (large white butterfly), and *Saturnia pavonia* (emperor moth), and again histologically found broadly similar results to Metalnikov with *G. mellonella*, indicating a universal lepidopteran response to mycobacteria (Hollande 1930). Infection of *G. mellonella* and *Lucilla sericata* (common green bottle fly) by *M. tuberculosis* strain H522 supports this view (Omodei et al. 1947). Cameron focused on the response of *G. mellonella* to *M. smegmatis* and *M. tuberculosis*, and again described similar findings to Feissinger, Hollande, Metalnikov, and Raedelli, but emphasized that he did not observe mycobacterial killing, but instead phagocytosis and survival within pericardial cells (Cameron 1934). The results contrasted with Metalnikov’s observation that ‘decomposition of the tubercle bacilli presenting a dark mass are finally absorbed by the pericardial cells’ (Metalnikov 1908). The assertion of Metalnikov that giant cell and capsule formation in *G. mellonella* in response to mycobacteria was analogous to human granuloma formation was rejected by Cameron, Feissinger, Hollande, and Redaelli, who all had the view that the response was, as Cameron summarized after reviewing the literature, ‘a nonspecific reaction’ (Cameron 1934).

The killing and nonkilling of mycobacteria as described by Metalnikov (1920) and Cameron (1934), respectively, may partly be explained by differences in strain and dose. Metalnikov (1920) describes using bacteria from ‘24–48 h culture on agar’ to prepare a ‘thick’ or ‘less dense’ emulsion in physiological water, and 1/40 or 1/80 of a cubic centimetre (1 cc = 1 ml) as the dose. It is, therefore, not possible to determine the doses used. In contrast, Cameron (1934) used a precise inoculum of 4 × 10^7^ CFU ml^−1^. It is also unlikely that the strain described as Koch’s bacillus by Metalnikov was *M. tuberculosis* as it grew on plates in 24–48 h, rather than 4–6 weeks as is typical. The lack of a strain designation in publications was commonplace: strain provenance and designation issues were known (Hastings and McCarter 1932, Tobie 1948). There had been concerns about the rapid growth of the widely used *M. tuberculosis* No 607 strain, also referred to as ‘Koch’s strain of the tubercle bacillus’, ‘Koch’s original strain’ or ‘Koch’s bacillus’ (Hastings and McCarter 1932, Tobie 1948). *Mycobacterium tuberculosis* No 607 and fast-growing ‘Koch’s bacillus’ were subsequently both shown to strongly resemble *M. smegmatis* (Hastings and McCarter 1932, Tobie 1948), and, subsequently, strain 607 was designated as *M. smegmatis* (Trevisan) Lehmann and Neumann ( = ATCC 607). It is, therefore likely, although not possible to determine, that the Koch’s bacillus used by Metalnikov and others was most likely *M. smegmatis* or another fast-growing mycobacterium. Despite the complications of strain and dose and interpretations of results, these early pioneers broadly found similar histological findings of the response of *G. mellonella* to mycobacteria, and the experiments provided a basis for future infection models a century later (see section immediately below).

### *Galleria mellonella* and *M. bovis* BCG interactions in the 21st century – the modern era

*Mycobacterium bovis* primarily causes tuberculosis in animals and is a source of substantial economic hardship for the worldwide bovine industry, with occasional zoonotic transmission to man, primarily in those working closely with cows (Livingstone et al. 2015). The live attenuated bacille Calmette–Guérin (BCG) vaccine, derived from a pathogenic *M. bovis* strain in the early 20th century, is the only one licenced to prevent tuberculosis and the most widely used vaccine in the world (Dockrell and Smith 2017). BCG shares a high degree of genetic similarity to *M. tuberculosis* and is commonly used as a surrogate for the latter (Garnier et al. 2003, Altaf et al. 2010, Harris et al. 2014, Turner et al. 2015, Painter et al. 2020) since it is usable at Biological Safety Level-2 (BSL-2).

The resurgence of *G. mellonella* in bacterial pathogenicity research (Tsai et al. 2016) led to the re-evaluation of *G.mellonella* as an infection model for mycobacteria using BCG as a surrogate for *M. tuberculosis* (Li et al. 2018). BCG was virulent for *G. mellonella* larvae, with an LD50 (the inoculum size that kills 50% of larvae) of 10^7^ CFU at 96 h. BCG doses >10^6^ CFU induced symptomatic disease with reduced larval motility, melanization, and wasting, but doses of <10^5^ CFU led to an asymptomatic and nonlethal outcome (Li et al. 2018). The observations by Li et al. (2018) contradicted the reported attenuation of *M. bovis* in early studies described in the section immediately above, which may be attributable to strain differences and/or the method to prepare inocula, e.g. the use of bacteria grown on agar plates (Cameron 1934) rather than those from more reproducible mid-log phase liquid cultures (Li et al. 2018). Visible colonies on solid growth media will contain a high proportion of stationary phase bacteria, which may be more susceptible to the *G. mellonella* innate immune system. Therefore, the lack of infection using 4 × 10^7^ CFU *M. bovis* in early studies is likely analogous to low-dose (<10^5^ CFU) infection using mid-log phase liquid cultures (Cameron 1934, Li et al. 2018). Similar discrepancies in *G. mellonella* between early and recent studies, including *Vibrio cholerae* (Bokhari et al. 2017) and *Streptococcus pneumoniae* (Cools et al. 2019), have been reported.

However, two recent studies reported a log difference in LD50 at 96 h for BCG infection in *G. mellonella* (Li et al. 2018, Bach-Griera et al. 2020). Such a difference could be attributable to the colony of *G. mellonella* and/or the BCG strain used. Commercial mass-reared larvae used by Li et al. (2018) are likely to be more heterogenous than those reared in-house used by Bach-Griera et al. (2020), and reasonable to assume population diversity may influence innate immunity and alter susceptibility to infection. The two studies also used different strains of BCG, Montreal (Li et al. 2018) and Connaught (Bach-Griera et al. 2020). These strains are closely related, but the *Mb3525c* to *Mb3527c* genes are deleted in the Montreal strain (Abdallah et al. 2015) and possibly contributed to the differences in virulence observed in *G. mellonella*. Numerous mutations including, deletions, insertions, and polymorphisms, have been identified between different BCG vaccine strains, particularly in virulence-related genes (Leung et al. 2008), and virulence variability between vaccine strains has been demonstrated in mice (Zhang et al. 2016). Genetic variability may also explain the different results obtained with BCG in *G. mellonella* in the 19th and 20th centuries with those in the modern era. Thus, while BCG is a surrogate for *M. tuberculosis*, researchers should be aware of its limitations, such as arising from genetic variability.

A dose of 10^7^ CFU of BCG led to a nonreplicative but persistent infection in *G. mellonella* over 2 weeks (Li et al. 2018, Asai et al. 2019b), indicating induction of innate immune antimicrobial activity supporting the observation of haemocyte-driven mycobactericidal activity reported by Metalnikov (1920). As BCG lacks the RD1 region required for efficient egress from phagocytes, bacilli are more susceptible to intracellular oxidative burst-mediated killing and enzymatic degradation (Liu et al. 2019). Nevertheless, the establishment of a persistent infection supports the claim of Cameron (1934) that *G. mellonella* cannot fully clear bacteria and persist throughout the lifespan of the moth. Recovery from the haemolymph of viable BCG 144 h postinfection also supports this view (Bach-Griera et al. 2020). Thus, it appears that recent BCG studies corroborate both Metalnikov and Cameron’s observations, and neither claim is definitive (i.e. killing and persistence can occur).

Proteomic analysis of the cell-free haemolymph found that BCG infection induced several AMPs, including cecropin A, a cecropin-D-like protein and gloverin (Asai et al. 2021). Protein expression was dose-dependent, with a higher CFU resulting in a higher differential in protein abundance. Detection of cecropin A was notable as a synthetic hybrid peptide containing the alpha-helical motif of cecropin A has antimycobacterial activity against BCG (Yue et al. 2020). Furthermore, cecropin A is known to possess immunomodulating activity like the human AMP LL-37, a key component of hCAP-18 treatment for tuberculosis (Torres-Juarez et al. 2015). These AMPs may have contributed to the reduction in BCG counts over time in *G. mellonella*.

**Figure 4.**
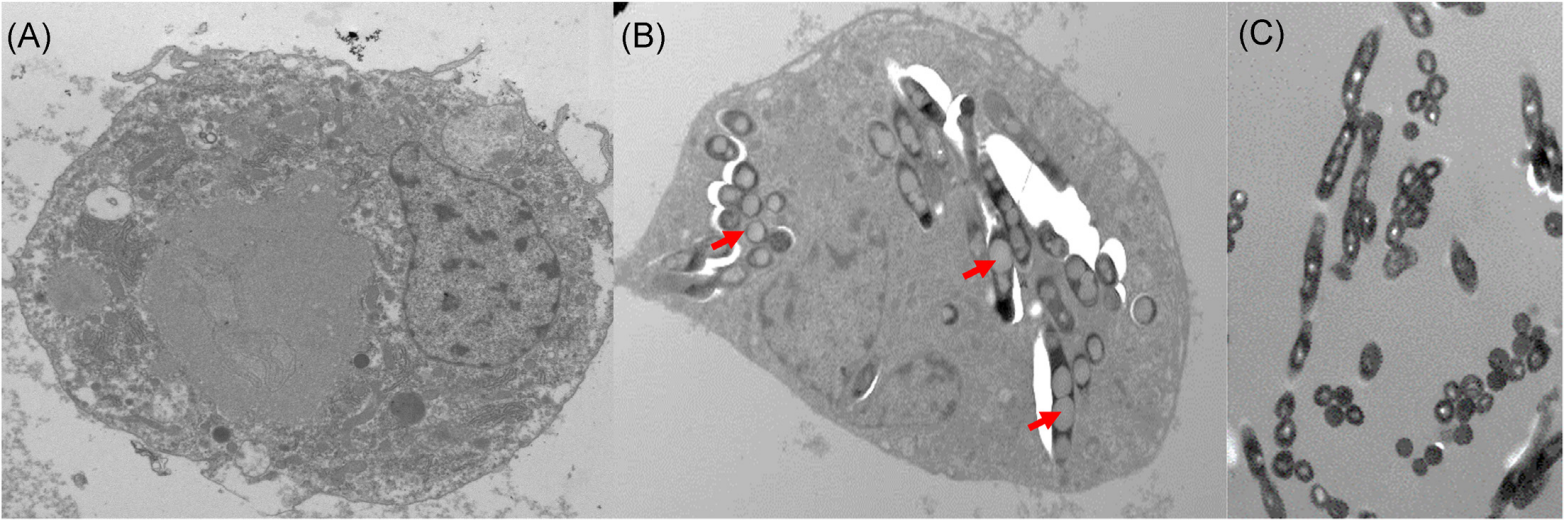
Transmission electron micrograph (TEM) of *G. mellonella* haemocytes. (A) TEM image of haemocyte recovered from naïve (uninfected) larva. (B) TEM image of haemocyte recovered from BCG-infected larva. Red arrows point to intracellular BCG showing accumulation of lipid fat bodies, a key phenotype indicating a metabolic switch from an active to a nonreplicative state. (C) TEM of BCG used as the inoculum for *G. mellonella* infection. Scale bars represent 500 nm. Adapted from Li et al. (2018) with permission.

After injection into *G. mellonella*, mycobacteria rapidly (as early as 30 min) associate with circulating haemocytes (Metalnikov 1920, Redaelli 1929, Hollande 1930, Cameron 1934). Transmission electron microscopy (TEM) confirmed this observation, showing membrane invagination typically associated with phagocytosis (Li et al. 2018). Furthermore, TEM revealed the formation of intracytosolic lipid inclusions (ILIs) within phagocytosed bacilli (Fig. 4). Similar observations have been reported for bacilli inside foamy macrophages present in granulomas, where ILIs are associated with nonreplicative and persistent populations of mycobacteria (Peyron et al. 2008, Russell et al. 2009). The lack of RD1 may force BCG to select preferentially for the expression of intracellular survival mechanisms (Jackson et al. 1989, Deb et al. 2009). These observations are further supported by the presence of the BCG protein diacylglycerol O-acyltransferase in the cell-free haemolymph of infected *G. mellonella* (Asai et al. 2021). Diacylglycerol O-acyltransferases are known to facilitate the formation of ILIs by accumulating triacylglycerol under intracellular stress and nutrient deprivation (Sirakova et al. 2006). Triacylglycerol is sourced from the cytoplasm of the haemocytes, which becomes fatty acid rich following immune activation (Péan et al. 2017, Skowronek et al. 2021).

**Figure 5.**
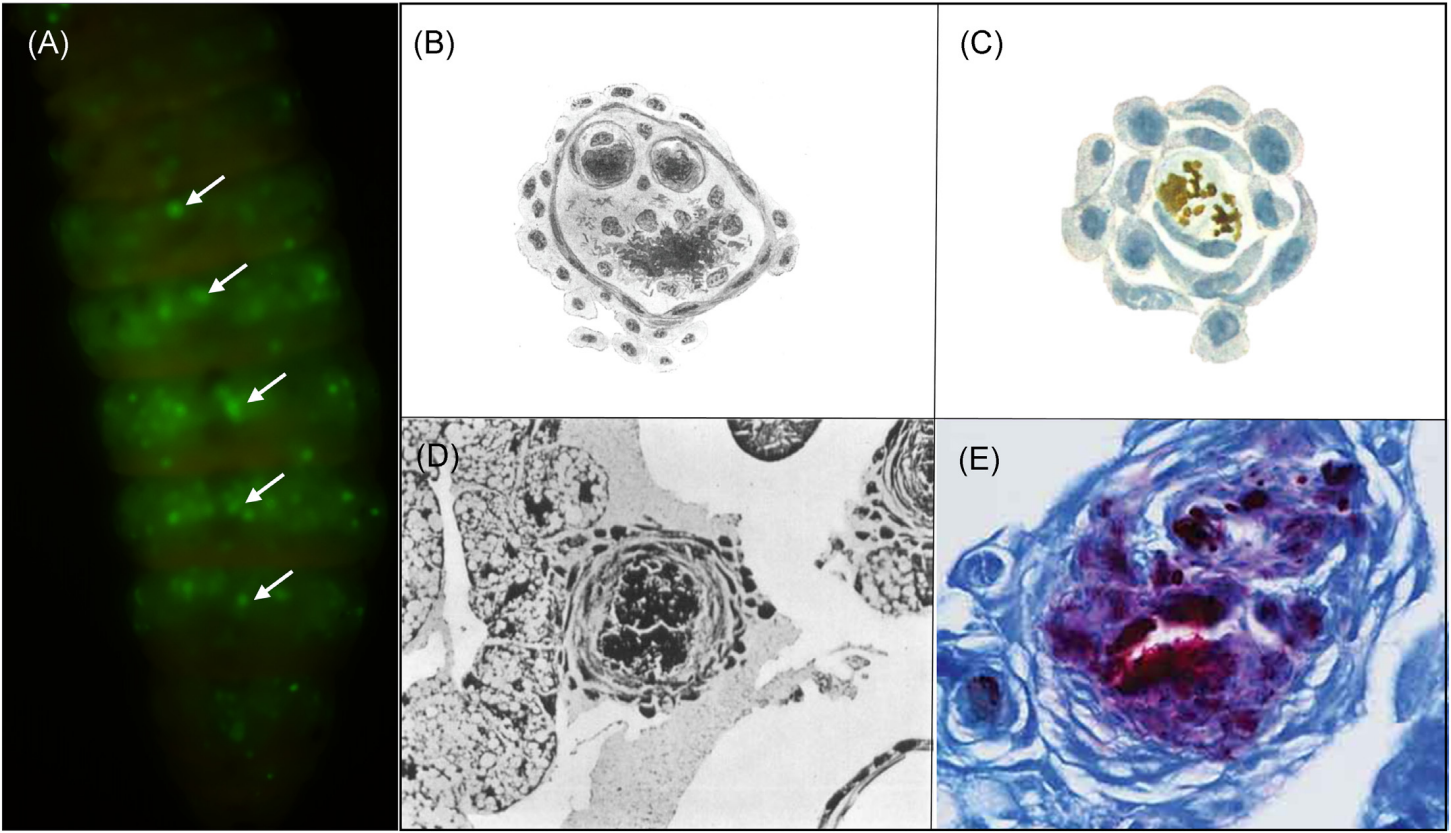
Visualization of mycobacterial-induced GLS in *G. mellonella*. (A) Infection is systemic with yellow fluorescence protein (YFP) expressing BCG establishing foci of infection throughout the larval cavity. (B)–(E) shows the consistency in results obtained by researchers over *c*. 100 years. Microscope images were sketched by (B) Metalnikov (1920), (C) Redaelli (1929), and photographed by (D) Cameron (1934) and (E) Li et al. (2018).

Histological analysis of *G. mellonella* infected with BCG also revealed an arrangement whereby haemocytes surround a central mass of infected cells, and the presence of bacterial aggregates throughout the larval body (Fig. 5A). These foci of infection resemble early-stage granulomas formed by innate immune cells in zebrafish larva (Davis et al. 2002) and were termed granuloma-like structures (GLS) in *G. mellonella* (Li et al. 2018). Haematoxylin and eosin (H&E) and Ziehl-Neelsen (ZN) staining of GLS identified that infected haemocytes had undergone cell necrosis, and BCG presence was confirmed by ZN-staining. Early studies reported similar structures to that reported by Li et al. (2018), often referring to GLS as nodules (Metalnikov 1920, Redaelli 1929, Hollande 1930, Cameron 1934) (Fig. 5B–D). GLS persist throughout the lifespan of the moth and are not digested or expelled from the body (Metalnikov 1920, Cameron 1934). Moreover, bacilli are recoverable from dissected GLS and can cause symptomatic disease in guinea pigs (Cameron 1934).

GLS development has been described as nodulation, a generic and nonspecific response involving plasmatocytes and granulocytes (analogous to neutrophils and macrophages) triggered to isolate nonself-material (Bergin et al. 2005). Nodulation begins with the attachment of granulocytes to an aggregate of microbes or infected cells (Jiravanichpaisal et al. 2006). Degranulation of granulocytes releases a plasmatocyte spreading peptide, which aggregates plasmatocytes leading to the formation of GLS (Kavanagh and Reeves 2004, Dubovskiy et al. 2016, Hillyer 2016). The putative defence protein Hdd11 (also called Noduler) is associated with the formation of GLS (Gandhe et al. 2007). Indeed, the proteome of cell-free haemolymph from *G. mellonella* infected with BCG identified a substantial increase in Hdd11 abundance of 18.3- and 310-fold at 48 h and 168 h postinfection, respectively, relative to the 0 h control (Asai et al. 2021). Currently, we lack the necessary tools for determining the precise development of GLS, and their relevance in this tuberculosis model remains unclear.

### *Galleria mellonella* and *M. tuberculosis* interactions in the modern era

Early *G. mellonella*–*M. tuberculosis* (and other mycobacteria) studies are hard to interpret because of lack of strain designation, difficulty in determining dose, and inadequate description of methods. In the modern era, well-characterized strains such as H37Rv, Erdman, M299, and CDC1551 are typically used in tuberculosis research. H37Rv is the most widely used and is phenotypically similar to the original isolate described by Koch (O’Toole and Gautam 2017) and the first strain to have its genome fully sequenced (Cole et al. 1998). The work with BCG presented by Li et al. (2018) highlighted the potential of *G. mellonella* as a model for tuberculosis but underlined the need for further evaluation using a well-characterized *M. tuberculosis* strain such as H37Rv.

To overcome the need for a BSL-3 facility essential for *M. tuberculosis* wild-type research, a double auxotroph (Δ*leuD* Δ*panCD*) of H37Rv (SAMTB) was constructed (Sampson et al. 2004). Lacking the ability to biosynthesize leucine (Δ*leuD*) and pantothenate (Δ*panCD*), SAMTB cannot survive without the supplementation of these metabolites. The double deletions substantially reduce the risk of reversion: thus, SAMTB has a BSL-2 designation. Unlike BCG, SAMTB retains RD1 and can synthesize and secrete antigens *in vitro* at near wild-type H37Rv levels (Mouton et al. 2019). Transcriptomic and proteomic comparisons of SAMTB and wild-type H37Rv found similar pathogenicity and responses to stress. Additionally, the antimicrobial resistance profiles and growth characteristics in macrophages of wild-type and SAMTB were comparable (Mouton et al. 2019).

In *G. mellonella*, SAMTB induced a virulent and lethal infection (LD50 of 2 × 10^7^ CFU at 96 h) with bacterial replication over the 7-day course. In contrast, SAMTB is avirulent in other experimental models, such as guinea pigs, mice, and nonhuman primates, as host leucine and pantothenate are not accessible or sufficient for replication and survival (Hondalus et al. 2000, Sampson et al. 2004, 2011). Thus, results suggest that leucine and pantothenate availability is sufficient for SAMTB replication and survival in *G. mellonella*, an insect, i.e. rich in these nutrients (Finke 2015, Killiny 2018), although their abundance may be insufficient to induce wild-type H37Rv level virulence as observed in the most optimal *in vitro* conditions (Mouton et al. 2019, Asai et al. 2020).

After studies using BCG and SAMTB, a *G. mellonella*–wild-type H37Rv (hereafter, referred to as H37Rv) model was evaluated (Asai et al. 2022). Using an infectious dose of 10^6^ CFU, the order of virulence, as measured by percentage survival, was H37Rv > SAMTB > BCG (Asai et al. 2022). These observations confirmed that *G. mellonella* is sensitive to different levels of mycobacterial virulence. The H37Rv study also demonstrated that the infectious dose can be reduced (from 10^7^ to 10^6^ CFU) and the study period extended (from 96 to 192 h), which better reflects study parameters of traditional tuberculosis animal models (Asai et al. 2022).

**Figure 6.**
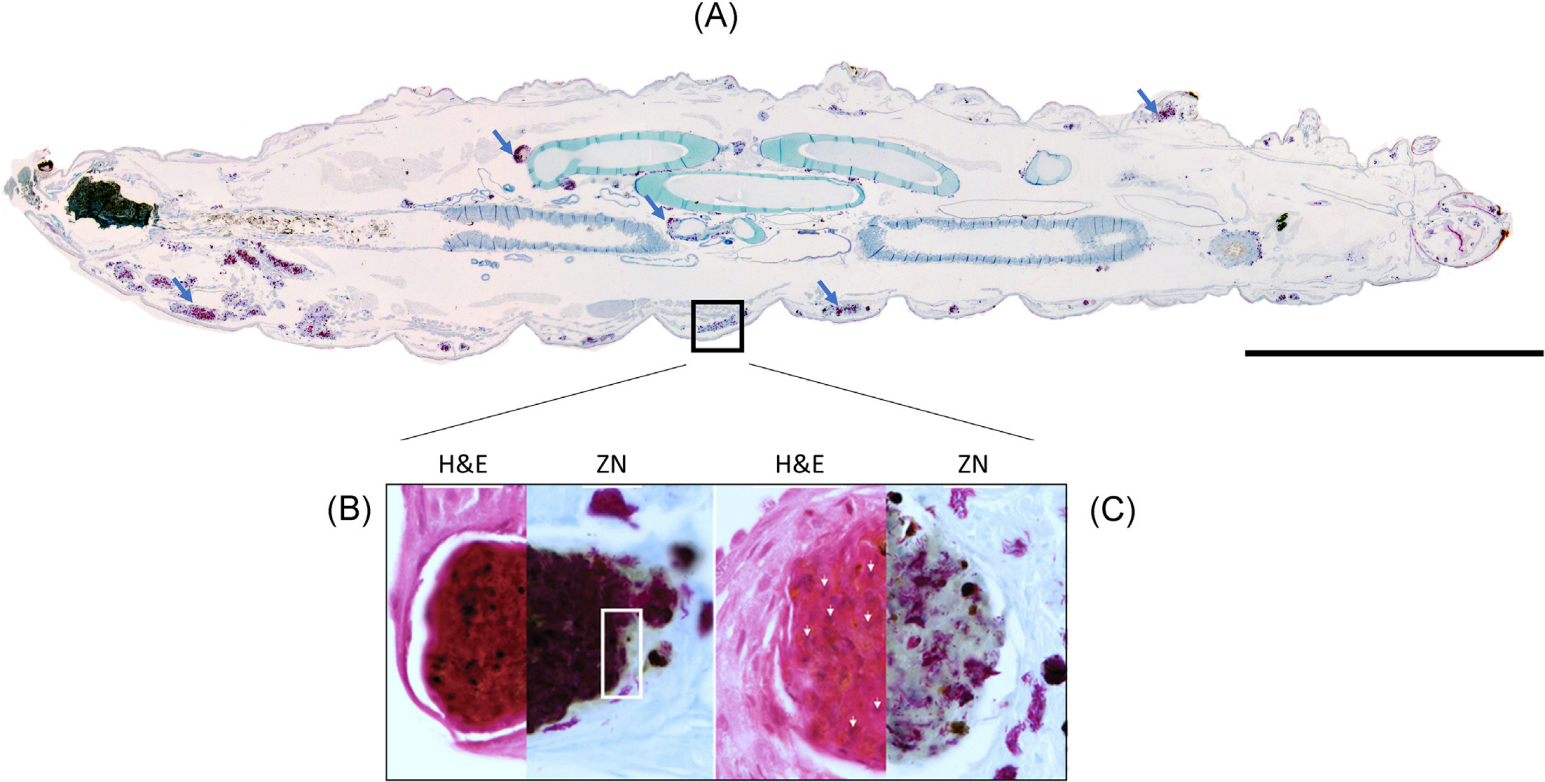
Loss of H37Rv ZN-staining in some *G. mellonella* GLS. (A) ZN-stained sagittal section of *G. mellonella* infected with *M. tuberculosis* H37Rv with GLS indicated by the blue arrows. Scale bar indicates 1 mm. (B) A representative GLS containing dense ZN-reactive material, with localized peripheral loss in ZN-reactivity indicated in the area enclosed by the white square. Foci of intense H&E staining most likely indicates host cell necrosis. (C) A representative GLS associated with a predominant loss of ZN-reactivity. In contrast to (B), non-ZN reactive masses had less intense H&E staining, and host cell nuclei were more easily distinguishable (as highlighted by the white arrows), likely indicating that host cell necrosis is low despite the presence of mycobacterial mass. Adapted from Asai et al. (2022) with permission.

The internalization of H37Rv by haemocytes was confirmed by TEM, as previously observed for BCG and SAMTB (Asai et al. 2020, 2022). However, unlike BCG, intracellular SAMTB and H37Rv lacked distinctive ILIs, likely reflecting active and replicative infection driven by RD1 (see ‘*M. bovis*’ section). Histopathology revealed the formation of GLS throughout *G. mellonella* (Fig. 6A), which were more abundant and larger with H37Rv and SAMTB infection compared to BCG. There were no visible differences in H37Rv, SAMTB, or BCG*-*induced GLS. However, a selection of GLS formed in response to H37Rv (Fig. 6B and C) and SAMTB contained ZN-negative clusters of bacilli. The loss of ZN-staining is a phenotype of persistent nonreplicative mycobacteria and is associated with short mycolic acid lipid chains (Bhatt et al. 2007). We speculate that a subpopulation of H37Rv and SAMTB are in a nonreplicative and persistent state within GLS. In contrast, BCG in GLS were all ZN-positive, despite many bacilli with large numbers of ILIs, another phenotype associated with nonreplicative and persistent bacilli. Further work is required to determine the extent of nonreplicative and persistent bacilli in H37Rv, SAMTB, and BCG-induced GLS.

The utility of *G. mellonella* as a comparative virulence screen for *M. tuberculosis* was determined by challenging larvae with H37Rv and isogenic Δ*phoP* and Δ*dosR* mutants, which were selected based on their known attenuation in some traditional mammalian models (Asai et al. 2022). As previously observed with severe combined immunodeficiency (SCID) mice (Martin et al. 2006), the Δ*phoP* mutant was attenuated in *G. mellonella*. In mammalian models, attenuation has been attributed to the impairment in ESAT-6 secretion, which prevents cell lysis, akin to the attenuation of BCG (Simeone et al. 2021). Additionally, Δ*phoP* can modulate the expression of crucial intracellular stress responses (hypoxia, persistence, and lipid metabolism) vital for mycobacterial survival (Gonzalo-Asensio et al. 2008). In contrast to Δ*phoP*, Δ*dosR* was hypervirulent in *G. mellonella* (Asai et al. 2022), contradicting reports of attenuation or indifference in nonhuman primates (Mehra et al. 2015), guinea pigs (Malhotra et al. 2004), rabbits (Converse et al. 2009), and immunocompetent mice (Converse et al. 2009, Gautam et al. 2015). However, hypervirulence in SCID mice has been reported (Parish et al. 2003). Both *G. mellonella* (Kavanagh and Reeves 2004) and SCID mice (Koo et al. 2009) lack a functional adaptive immune system, which in SCID mice was shown to be necessary to overcome the 8–10-fold higher Δ*dosR* bacillary load (Parish et al. 2003).

### *Galleria mellonella*–MTBC infection models as a screen for antimycobacterial compounds

Tuberculosis treatment relies on a limited selection of antimycobacterial compounds, and the efficacy of these compounds continues to dwindle with the emergence and spread of antibiotic-resistant *M. tuberculosis* (Mizrahi and Warner 2019). Despite the threat of tuberculosis on public health, only three novel compounds for clinical use have been approved in the past five decades (World Health Organization 2020). The use of *G. mellonella* as a drug screen has become increasingly popular as a bridge between *in vitro* and *in vivo* mammalian models by producing efficacy data for treatments in a more ethical, economical, and timely manner (Cutuli et al. 2019). The first use of *G. mellonella* to test antimycobacterial antibiotics was in the 1940s with the pioneering studies of Morrellini and Cattaneo. An excellent comprehensive review describes their published and unpublished work (Ferrari and Barbaro 1964); a summary only is given here. In brief, after confirming that their preferred strain *M. tuberculosis* H522 replicated in *G. mellonella* (Omodei et al. 1947) they tested, in a proof-of-concept study, whether larvae could assess antimicrobial activity *in vivo*, using the recently discovered antimycobacterial agent streptomycin and inactive penicillin as a control agent (Ferrari and Barbaro 1964). Antibacterial treatment was initiated 2–3 days after infection, with further injections given on alternate days. Streptomycin prevented infection by injection (not orally), but there was no effect of penicillin mimicking results in humans and validating the model (Ferrari and Barbaro 1964). Subsequently, they showed that para-aminosalicylic acid – another promising antimycobacterial agent *in vitro* – was active against H522 both by injection and when given orally. This work led to further studies identifying isonicotinic acid hydrazide (isoniazid, INH), terramicin (oxytetracycline), and a thiosemicarbazone as having antimycobacterial activity in *G. mellonella* against H522 and H37Rv (Morellini and Cattaneo 1953, Ferrari and Barbaro 1964). The thiosemicarbazone of para-aminosuccinyl–benzaldehyde (TBI/698) was also active in *G. mellonella* (Ingrao and Belli 1951), although some thiosemicarbazone derivatives, contrary to *in vitro* results, were not (Morellini and Cattaneo 1953). In addition, an H522 streptomycin-resistant clone remained sensitive to para-aminosalicylic acid, terramicin, and thiosemicarbazone in *G. mellonella*, i.e. there was no cross-resistance between antibiotics *in vivo* (Morellini et al. 1953). These experiments established the potential of using *G. mellonella* as a screen for compounds with anti-*M. tuberculosis* activity *in vivo*.

*Galleria mellonella* was subsequently re-evaluated as a drug screen for antimycobacterial compounds using BCG, SAMTB, and H37Rv infection models in the modern era (Asai et al. 2019a, 2020, 2022). *Galleria mellonella* infected with MTBC were treated with a first-line antimycobacterial: ethambutol (ETH), INH, pyrazinamide (PZA), or rifampicin (RIF), or the second-line antimycobacterial: moxifloxacin (MOX). Drug screening used compounds at doses recommended for the treatment of adult tuberculosis relative to the body mass of the larvae (∼200 mg). The above *G. mellonella* studies, at least in part, also used mycobacterial reporter strains enabling the rapid determination of *in vivo* load following treatment: luminescence was used as a relative measure for CFU (Asai et al. 2019b, 2020, 2022). All compounds improved larval survival relative to mock PBS-treated controls, with INH and RIF being the most effective in reducing *in vivo* mycobacterial load. ETH and MOX only had efficacy at 10x the recommended clinical dosage. Despite higher larval survival, PZA did not significantly reduce *in vivo* mycobacterial load, even at 10x the recommended clinical dosage (Asai et al. 2019a, 2020). Furthermore, the lead time to treatment following infection (1 or 72 h postinfection) did not affect the overall efficacy of ETH, INH, PZA, or RIF (Asai et al. 2019a, 2020, 2022). Overall, the drug efficacies of ETH, INH, MOX, and RIF reported in *G. mellonella* were comparable to those found in mice (Nikonenko et al. 2004, Driver et al. 2012, Jia et al. 2005, Zimmerman et al. 2017). In mice, e.g. INH and RIF were the most effective (Driver et al. 2012). ETH, INH, MOX, and RIF had potent antimicrobial activity at dosages similar to those used in *G. mellonella* (Nikonenko et al. 2004). MOX was more potent than ETH and RIF, which had similar activity levels. However, it is hard to compare the results in mice with those in *G. mellonella* since studies with the former typically involve multiple dosing (Nikonenko et al. 2004, Driver et al. 2012). In the *G. mellonella*-H37Rv model, ETH had the least potent activity relative to INH and RIF, with CFUs supporting a bacteriostatic activity similar to those reported in mice (Asai et al. 2022, Jia et al. 2005, Zimmerman et al. 2017).

The lack of PZA activity in *G. mellonella* could be for many reasons. BCG is intrinsically resistant to PZA as it lacks *pncA*, which encodes the pyrazinamidase required for the conversion of prodrug PZA into active pyrazinoic acid (POA) (Ritz et al. 2009). SAMTB is also resistant due to the loss of *panD*, which encodes aspartate decarboxylase, a vital component of the pantothenate biosynthesis pathway and a known target of POA (Gopal et al. 2017, Mouton et al. 2019). The lack of PZA activity against H37Rv was hypothesized to result from host pantothenate availability concealing any inhibitory activity by PZA/POA on aspartate decarboxylase (Asai et al. 2022). Nevertheless, larvae treated with PZA showed improvements in survival despite the lack of *in vivo* mycobacterial reduction (Asai et al. 2019b, 2020, 2022). Disruption of mycobacterial phthiocerol dimycocerosate synthesis by PZA treatment and induction of a sublethal *in vivo* phenotype has also been proposed (Gopal et al. 2016). For ETH and MOX, the lack of antimycobacterial activity at the recommended clinical dosage likely reflects insufficient drug reaching the site of infection, illustrating the lack of data on the pharmacokinetic and pharmacodynamic properties of compounds in *G. mellonella*.

Tuberculosis treatments typically comprise a cocktail of compounds given over a period of 6 months or more (World Health Organization 2017). ETH, INH, MOX, and RIF combinations were tested in *G. mellonella* infected with BCG or SAMTB, however, no combination was better than INH or RIF alone (Asai et al. 2019a, 2020). The lack of improvement in antimycobacterial efficacy is not discouraging since the purpose of combination therapy is to minimize the development of drug resistance and not necessarily for synergistic activity (World Health Organization 2017). Multiple doses of INH, but not RIF, significantly improved antimycobacterial activity in *G. mellonella* infected with SAMTB or BCG (Asai et al. 2019a, 2020).

An integral part of drug development is to determine the toxicity of novel compounds, and studies have shown that *G. mellonella* is an excellent predictor of toxicity (Allegra et al. 2018, Suay-García et al. 2019). Studies with *G. mellonella* to either verify the lack of toxicity of existing antimicrobials, e.g. ETH, INH, MOX, PZA, and RIF (Asai et al. 2019a) or to assess novel antimycobacterial toxicity have also been reported (Briffotaux et al. 2022, Di Blasio et al. 2023). A novel MmpL3 (mycolic acid transporter) inhibitor was evaluated for both toxicity and efficacy in *G. mellonella* infected with BCG and shown to be nontoxic and have efficacy near that of RIF (Briffotaux et al. 2022). Similarly, the toxicity and efficacy of bola-amphiphiles were assessed in uninfected and *M. tuberculosis*-infected *G. mellonella* larvae, respectively (Di Blasio et al. 2023). They were nontoxic, but efficacy *in vitro* and *in vivo* was contradictory. Bola-amphiphiles with short-chain lipids were the most active *in vitro* but exacerbated the infection *in vivo*; those with long-chain lipids were less active *in vitro* but had good efficacy *in vivo* (Di Blasio et al. 2023). As a model with considerable value as a bridge between *in vitro* and more complex *in vivo* screening, using *G. mellonella* to assess toxicity and efficacy simultaneously prior to screening in mammalian models will help reduce the number of animals used. In addition, such models will yield faster early-stage results accelerating translation into clinical research (Hunter 2023). A possible future use of *G. mellonella* is in screening for transgenerational epigenetic-mediated side effects of antimycobacterial compounds, such as described for psychoactive drugs in the *Tribolium castaneum* (red flour beetle) model (Bingsohn et al. 2016).

## Nontuberculous mycobacteria (NTM)

NTM are a group of ubiquitous environmental mycobacteria that are opportunistic pathogens, generally categorized into rapidly growing mycobacteria (RGM) or slowly growing mycobacteria (SGM) (Ratnatunga et al. 2020). Four distinct clinical manifestations of NTM infection have been described in humans; chronic pulmonary disease, systemic infection in immunocompromised individuals, skin and soft tissue infection, and superficial lymphadenitis, with the majority (80%–90%) manifest as a pulmonary infection (Griffith et al. 2007). *Mycobacterium abscessus* [belonging to the *M. abscessus* complex (MABC)], *Mycobacterium kansasii*, and *M. avium* [belonging to the *M. avium* complex (MAC)] are the most common causes of human NTM disease. NTM infections are more prevalent amongst individuals with pre-existing congenital or acquired pulmonary diseases, such as chronic obstructive pulmonary disease (COPD) and cystic fibrosis, and in the healthcare-associated setting. The rate of NTM incidence in populations with no predisposing conditions is increasing alarmingly (Moon et al. 2020). Treatment of NTM is generally challenging as mycobacteria are intrinsically resistant to many antibiotics (Johansen et al. 2020). However, most NTM are susceptible to macrolide-based antibiotics (e.g. azithromycin or clarithromycin), and combination treatment with macrolides minimizes further development of drug resistance. Like tuberculosis, the treatment regimen is long (lasting up to 18 months), costly, and associated with unpleasant side effects (Saxena et al. 2021).

## M. abscessus complex (MABC)

The MABC group of NTM consists of *M. abscessus* sub-spp. *abscessus, Mycobacterium massiliense*, and *Mycobacterium bolletii*, and considered the most pathogenic among the RGM (Victoria et al. 2021). Members of the MABC cause pulmonary infections: the most common in the RGM and prevalent in individuals with cystic fibrosis (Victoria et al. 2021). MABC pathogenicity is associated with two colony phenotypes: rough or smooth (Fregnan and Smith 1962). Compared to the smooth phenotype, the rough phenotype is associated with a more aggressive and destructive infection (Bernut et al. 2014). The rough phenotype can promote apoptosis during infection, releasing large extracellular cords of bacilli, which are immune to phagocytosis by macrophages. The cords of bacilli can further propagate by invading surrounding tissue or through extracellular replication (Bernut et al. 2014). The switch from smooth to rough phenotype is associated with a loss in glycopeptidolipid on the outer surface of the cell wall, which exposes a proinflammatory lipoprotein, which induces a more aggressive and destructive infection relative to the smooth phenotype (Nessar et al. 2011). In contrast, persistent infection, likely due to lower proinflammatory propensity, is associated with the smooth phenotype (Ruangkiattikul et al. 2019). The factors that drive switching between rough and smooth phenotypes are currently unknown.

The use of MABC in *G. mellonella*, i.e. *M. abscessus* sub-spp. *abscessus*, was first described using the smooth reference strain ATCC 19977 and a rough clinical isolate (Meir et al. 2018). An RGM nonpathogenic *M. smegmatis* strain was used as an avirulent control (Meir et al. 2018). Infections resulted in virulent and lethal outcomes in the order of rough > smooth > *M. smegmatis* (Meir et al. 2018). These observations were supported by screening more *M. abscessus*, with a rough *M. abscessus* sub-spp. *massiliense* strain being the most virulent (García-Coca et al. 2019). Collectively these data align with clinical observations where the rough phenotype causes a more severe infection leading to higher morbidity (Caverly et al. 2015). *Mycobacterium abscessus* sub-spp. *abscessus*-infection *in vivo* was proliferative, with CFU increasing by 3.5 logs over 6 days (Meir et al. 2018). Histological analysis of larvae infected with *M. abscessus* revealed the formation of GLS in fat tissue. However, whether the rough or smooth phenotype affected GLS formation was not discussed.

The application of the *G. mellonella*–*M. abscessus* model as a drug screen was evaluated using amikacin (AMK), azithromycin, levofloxacin, linezolid, meropenem, piperacillin, and tigecycline. Only treatment with AMK, azithromycin, or meropenem significantly improved larval survival (Meir et al. 2018). Treatment equated to two daily doses for treatment of adult MABC infection (relative to body mass of larvae) and antibiotics were administered at 24 and 48 h postinfection. Drug efficacy was assessed using a bioluminescent smooth *M. abscessus* strain and an imaging system (Meir et al. 2018). This approach enabled the quantification of drug efficacy within individual larva without the necessity for killing and homogenization, typically used for CFU enumeration. Treatment with AMK, meropenem, and tigecycline, but not azithromycin, led to significant reductions in bioluminescence (Meir et al. 2018). The most efficacious compounds were AMK and meropenem, which correlate with the recommended treatment guidelines (Griffith et al. 2007, Floto et al. 2016). CFU enumeration indicated significant synergistic activity of AMK and meropenem with tigecycline against the rough strain, but there was no discussion of the implications.

## *Mycobacterium marinum, Mycobacterium fortuitum*, and *Mycobacterium aurum*

*Mycobacterium marinum* is an SGM that causes piscine mycobacteriosis (more commonly known as fish tuberculosis). As an opportunistic pathogen, *M. marinum* infection is prevalent in fishermen, often referred to as ‘fish tank granuloma’ or ‘fish handler’s disease’ (Hashish et al. 2018), and clinically is typically a superficial skin infection characterized by granuloma formation and lymphangitis. *Mycobacterium fortuitum* is an RGM, with bone/joint, ocular, and skin infections being the most common manifestations; pulmonary infection is uncommon (Griffith et al. 2007). Beyond their significance as opportunistic pathogens, *M. marinum* and *M. fortuitum* are popular surrogates for *M. tuberculosis in in vitro* and *in vivo* studies. *Mycobacterium marinum* is widely used to model tuberculosis in zebrafish (Swaim et al. 2006, Takaki et al. 2013), and *D. melanogaster* (Dionne et al. 2003, Oh et al. 2013), which are not compatible with *M. tuberculosis* as these model hosts cannot be grown at 37°C (Pype et al. 2015, Marshall and Dionne 2022). *Mycobacterium marinum* is an excellent surrogate for *M. tuberculosis* in both zebrafish and *D. melanogaster* models, including for studies of intracellular adaptation (lipid metabolism), complex presentations such as tuberculous meningitis, and the transparency of zebrafish allows real-time visualization of events leading to the formation and maintenance of granulomas, and both models are genetically tractable (Tobin and Ramakrishnan 2008, van Leeuwen et al. 2014, Péan et al. 2017, Chen et al. 2018, Marshall and Dionne 2022). Genetically, 85% of the orthogonal coding sequence between *M. marinum* and *M. tuberculosis* is shared, including RD1 (Stinear et al. 2008). The fast-growing nature of *M. fortuitum* enables rapid data generation, and drug screening data have been used as a barometer of potential activity in *M. tuberculosis* (Renau et al. 1995, 1996, Gillespie et al. 2001, 2005). *Mycobacterium aurum* is an RGM typically considered a commensal, although rare cases of infection in immunocompromised individuals have been reported (Honarvar et al. 2012). *Mycobacterium aurum* has also been used as a surrogate for *M. tuberculosis* for *in vitro* drug screening of antimycobacterial compounds (Gupta et al. 2009, Gupta and Bhakta 2012).

Entwistle and Coote (2018) compared *G. mellonella*–*M. aurum*/*M. fortuitum* models of infection, their first description, with *M. marinum*. In total, three *M. marinum* strains – R356933F, M, and NCTC 2275 – were screened in *G. mellonella* for virulence over 144 h using a range of inocula. R356933F was the most virulent. Similarly, *M. fortuitum* NCTC 10394 was more virulent than NCTC 8573. *Mycobacterium aurum* NCTC 10437 was avirulent in *G. mellonella*, including at high doses. Collectively, the order of virulence between the three species was *M. marinum* > *M. fortuitum* > *M. aurum* (Entwistle and Coote 2018). A separate study identified that rough *M. fortuitum* induced a more lethal infection in *G. mellonella* than smooth strains (García-Coca et al. 2019). An initial reduction of *in vivo* mycobacterial CFU was reported for *M. fortuitum* and *M. marinum* (Entwistle and Coote 2018), although the latter established a proliferative infection over 144 h. In contrast, *M. marinum* numbers declined, presumably due to the inability to grow at 37°C (Collins et al. 1985, Tobin and Ramakrishnan 2008).

The applicability of *G. mellonella*–*M. fortuitum*/*M. marinum* infection models for screening drugs was investigated by treating larvae 2 h postinfection with AMK, ciprofloxacin (CIP), ETH, INH, or RIF (Entwistle and Coote 2018). AMK and ETH were the most and least efficacious drugs for both bacteria. CIP was particularly efficacious against *M. fortuitum*; INH for *M. marinum*. Despite improvement in survival, treatment of *M. fortuitum-*infected larvae with AMK or CIP did not significantly reduce mycobacterial burden over 144 h. In contrast, AMK or INH treatment significantly reduced mycobacterial burden in *M. marinum*-infected larvae, but only for the first 96 h. Combinations of 3–4 drugs increased larval survival, treatment with AMK + ETH + INH + RIF being the most effective. However, outcomes of *G. mellonella*–*M. marinum*/*M. fortuitum*interactions in the context of efficacy against *M. tuberculosis* were not discussed. The synergy of combinations was attributed to an increased abundance of phagocytic haemocytes in response to AMK and ETH, in addition to the basal antimycobacterial activities of each compound (Entwistle and Coote 2018). Activation of the innate immune response following injection of an antimicrobial alone has been reported previously in *G. mellonella* (Kelly and Kavanagh 2011).

## Mycobacterium kansasii

*Mycobacterium kansasii* is a slow-growing NTM, i.e. an increasingly recognized cause of chronic pulmonary disease in humans, especially amongst those with bronchiectasis, COPD, HIV, and previous mycobacterial disease (Akram and Rawla 2022). The bacterium was first isolated from patients with tuberculosis-like pulmonary disease in 1953 (Buhler and Pollak 1953). Incidence is underreported, especially in areas with high tuberculosis–HIV burdens, since clinical features are like those caused by *M. tuberculosis*, and there is a lack of specific diagnostics (Budell et al. 2020). Standard treatment for pulmonary *M. kansasii* infection is like that for *M. tuberculosis*, i.e. ETH, INH, and RIF (the key antimicrobial) for 12 months; resistance is of increasing concern (DeStefano et al. 2018). Historically, *M. kansasii* has been categorized into subtypes I–VII, subtype I being the most prevalent in humans, and recent whole genome sequence-based analyses have indicated that subtypes II–VI should be considered closely related but different species (Jagielski et al. 2019, Tagini et al. 2019, Guan et al. 2020, Luo et al. 2021). These analyses also confirmed the close genetic relationship of *M. kansasii* with *M. tuberculosis. Mycobacterium kansasii* can survive in the environment, especially in water, where it can form biofilms (Schulze-Robbecke and Fischeder 1989), and it has been suggested as an exemplar model organism to study the evolution of the switch from an opportunistic environmental to an obligate host-restricted intracellular pathogen (Wang et al. 2015).

The basic biology and mechanisms of the pathogenicity of *M. kansasii* are relatively unknown. Gene function studies are commonly used to gain such knowledge, but the lack of genetic tools and accessible infection models has limited progress (Budell et al. 2020). To address these limitations, a ϕMycoMarT7 phagemid-based transposon mutagenesis system for producing *M. kansasii* mutants, and a *G. mellonella*–*M. kansasii* infection model to compare the virulence of wild-type and isogenic transposon mutants, were developed (Budell et al. 2020). Over 12 000 *M. kansasii* mutants were screened for defects in microcolony formation, the rationale being that such mutants were also likely to be defective in biofilm formation, a known important environmental survival trait and a contributor to mycobacterial pathogenesis (Aldridge et al. 2014, Chakraborty and Kumar 2019). Mutant 13D6 was selected for further study, being smaller and having an uncharacteristic smooth glistening appearance, and subsequently shown to have an insertion in a gene encoding a small RNA of unknown function called *B11*. 13D6 was defective in biofilm formation. Wild-type and 13D6 were compared in the newly developed *G. mellonella* infection model, whereby larvae were injected with 10^7^ CFU and incubated at 25°C or 37°C in the dark. Larvae were killed at 37°C to the same extent by wild-type and 13D6: there was no significant killing at 25°C, attributed to the ‘less favourable’ bacterial replication at this lower temperature. The conclusions were that disruption of *B11* transcription in 13D6 had no impact on virulence in *G. mellonella* and that the findings provided the first demonstration of the susceptibility of *G. mellonella* larva to *M. kansasii* (Budell et al. 2020). Further work is required to determine whether the model is usable with different strains and whether the *G. mellonella* host response is similar to or different from other mycobacteria. The results also suggest that *M. kansasii* biofilm formation is not required for pathogenicity in *G. mellonella*.

## *Mycobacterium leprae* and *M. lepraemurium*

*Mycobacterium leprae* is the causative agent of leprosy, a highly contagious chronic infectious disease that affects the skin and peripheral nerves (Avanzi et al. 2016). Leprosy is also called Hansen’s disease after the Norwegian Gerhard Armauer Hansen, who discovered it in 1873. No one has been able to culture colonies of this strict obligate intracellular bacterium *in vitro* and, in addition to humans, the nine-banded armadillo (Truman 2005, Hess and Rambukkana 2019) and red squirrel (Avanzi et al. 2016) are naturally susceptible to multibacillary-like disease. *Mycobacterium leprae* also grows in the foot pad of nude mice (Adams et al. 2012), zebrafish (Madigan et al. 2017), and in macrophages cocultured with autologous human peripheral blood mononuclear cells (Wang et al. 2013), all of which involve the formation of granulomas, like those found in the human lepramatous form.

*Galleria mellonella* larvae have been evaluated for their potential to propagate strains that cause leprosy. Material from nodules, nasal mucus, and the blood of leprosy patients was injected directly or after homogenization into the body cavity of *G. mellonella* larvae (Signorini and Terni 1950). In 5/28 and 1/9 larvae injected with nodules and nasal mucus, respectively, ZN-stained bacilli were seen at autopsy 2–11 days postinfection. However, attempts to transmit the infection to other larvae were unsuccessful (Signorini and Terni 1950), as was cultivation (Terni and Signorini 1950). Thick emulsions of *M. lepraemurium* (also called Stefansky’s bacterium), which causes leprosy in mice and rats (Stefansky 1903) and is often used as a surrogate for *M. leprae*, were injected into *G. mellonella* larvae and bacteria were phagocytosed and engulfed in giant cells (Metalnikov and Toumanov 1923). Phagocytosis occurred within the haemolymph between 30 and 120 min but was considered weak. A total of 3–4 weeks postinfection, in both chrysalids and butterflies, large clusters of leucocytes and giant cells filled with bacteria were ZN-stained, indicative of the presence of mycobacteria. The authors concluded that there was little toxicity of the leprosy bacilli or their toxins for *G. mellonella*, and a ‘kind of symbiosis’ between the insect and the bacteria formed. *Galleria mellonella* larvae were also infected with *M. lepraemurium* and kept at 4°C: the bacterium was isolated from *G. mellonella* at 2.5 months and infectious for rats, indicating that *M. lepraemurium* can survive in *G. mellonella* (Signorini and Gargani 1952). Thus, in the case of *M. lepraemurium*, there is clear evidence that bacteria can remain viable in *G. mellonella* at 4°C. No such data exists with *M. leprae* and is complicated by infected *G. mellonella* larvae having been kept at 37°C: a suboptimal temperature for bacterial survival, and no culture methods being available.

## *Mycobacterium indicus pranii* (MIP)

The MAC comprises *M. avium* and *Mycobacterium intracellulare*, which cause disseminated and pulmonary infections (Inderlied et al. 1993). Such bacteria are found in dust, soil, and water but cause infection when inhaled or swallowed, especially in the immunocompromised, or those with conditions such as COPD or cystic fibrosis. MIP is typically considered a nonpathogenic member of the MAC, although it can cause human pulmonary infections (Kim et al. 2015). More recently, based on whole genome sequence analyses, it has been suggested that MIP should be classified as *M. intracellulare* subsp. *intracellulare* genovar *paraintracellulare* (Tateishi et al. 2021). Interest in MIP stems from its use as an immunotherapeutic to prevent or treat leprosy (Sharma et al. 2017) and tuberculosis (Saqib et al. 2016), but also as a surrogate for *M. tuberculosis*. Three studies have used the *G. mellonella*–MIP infection model to assess candidate anti-*M. tuberculosis* agents (Rwegasila et al. 2016, 2018, Erasto et al. 2018). The first evaluated formulations of the polyphenol panchovillin (PANV) produced by many plants, including *Erythrina schliebenii*, a species endemic to but only found in Tanzania, where the group were based (Rwegasila et al. 2016). PANV was chosen as a representative polyphenol as it had known activity against *M. tuberculosis* H37Rv (Nyandoro et al. 2017). Shrimp chitin was the chitosan (CS) source, and nanocomposites were synthesized by inotropic gelation with tripolyphosphate (TPP). MIP-infected larvae were melanized after 10 min, and all died at 24 h of incubation at 37°C. There was no statistical difference in survival between PANV and PANV-CS/TPP treated larvae at 48 h postinfection, in contrast to 24 h where PANV prolonged survival. Larvae injected only with CS/TPP were equivalent in survival and histology to uninfected controls. Although not stated, treatment most likely was given soon after MIP larval infection. A similar study, with the *N*-cinnamoyl tetraketide derivative toussaintine A (TA) isolated from *Toussaintia orientalis* Verdic (*Annonaceae*), another Tanzanian-endemic plant, has been published (Rwegasila et al. 2018). TA was known to have *in vitro* activity against *M. tuberculosis* H37Rv (Nyandoro et al. 2015). Similar results were found in *G. mellonella* to those for PANV, with a statistical difference in survival of MIP-infected larvae between free TA and TA-CS/TPP treated groups at 48 h but not 24 h. Finally, an alkaloid hydroxybenzylisoquinoline (also known as higenamine) was isolated from the leaves of *Aristolochia brasiliensis* Mart. & Zucc (*Aristolochiaceae*), another native plant to Tanzania (Erasto et al. 2018). The plant has a history of medicinal use in Tanzania for chronic and persistent coughs and as a herbal supplement for immunocompromised patients, including those with AIDS and tuberculosis. The rationale for MIP use was that *in vitro* resistance to INH made it a good surrogate for resistant *M. tuberculosis*. A single dose of higenamine given to larvae resulted in 80% survival, compared to 100% deaths in the untreated group. Further experiments showed that survival was dose-dependent. In contrast to INH resistance *in vitro*, MIP was highly susceptible to the drug in *G. mellonella* larvae. The explanation for the difference in INH activity *in vitro* and *G. mellonella* is unknown. They claimed that ‘the use of *G. mellonella* as a host for mycobacterial infections has never been documented elsewhere, thus it is herein reported for the first time’ despite the same group publishing a similar model earlier (Rwegasila et al. 2016). Clearly, given the work done in the early part of the 20th century, the claim does not stand, but is, to our knowledge, the first to describe a *G. mellonella*–mycobacteria infection model in the modern era.

In summary, although only used in a few studies, the *G. mellonella*–MIP infection model has shown potential in evaluating natural products. Further work is required to define the infectious process and the true extent of its ability to predict the efficacy of antimycobacterial activity to treat human tuberculosis.

## *Mycobacterium avium* subsp. *hominissui*s (MAH)

MAH is a member of the MAC found in dust, soil, straw, and water but can also cause opportunistic infections in mammals, including cattle, humans, and pigs (Scherrer et al. 2018). As with other members of the MAC, the risk of human infection is highest in the immunocompromised, e.g. HIV-infected, or those with bronchiectasis, COPD, or cystic fibrosis: infections in immunocompetent hosts can also occur (Thomson and Yew 2009). Disseminated infections are typically associated with the gastrointestinal and respiratory routes in immunocompromised hosts or pigs and immunocompetent hosts, respectively. High levels of antibiotic resistance have been found, particularly in human pulmonary isolates (Uchiya et al. 2018). The bacterium, because of its importance in causing human infection, similarities in virulence to *M. tuberculosis* (e.g. proliferation in macrophages and hijacking of phagosomal trafficking), and high antibiotic resistance, has been used as a surrogate in antimycobacterial screens (Moreira et al. 2016, Yang et al. 2017). Kirubakar et al. (2018) evaluated the comparative virulence of a wild-type MAH strain, originally isolated from a patient with HIV infection (Horan et al. 2006), a *lysX* mutant, and a complemented strain. In MAH, *lysX* encodes a lysyl-transferase-lysyl-tRNA synthetase, previously associated with AMP resistance (Kirubakar et al. 2018), similar to that reported for the *M. tuberculosis* ortholog (Maloney et al. 2009). *Galleria mellonella* larvae were infected with a dose of 10^6^ CFU and followed for 18 days, with death occurring predominantly from day 12 postinfection. The *lysX* mutant was the most virulent: all infected larvae had died at 18 days, contrasting with 40% and 65% in the wild-type and complemented groups, respectively. At 10 days postinfection, an ∼100-fold increase in CFU of the *lysX* mutant compared to wild-type and complemented strains was found. The results were consistent with an improved ability of the *lysX* mutant to multiply within human monocyte-derived cells (Kirubakar et al. 2018). The authors concluded that the *lysX* mutation causes a cellular metabolic status similar to that found in *M. tuberculosis*, facilitating intracellular survival, and hypothesized that observed changes in surface glycopeptidolipids might be involved (Kirubakar et al. 2020). Although it is comparatively unusual for a mutation in a bacterial gene to increase virulence, as in the case of the *lysX* mutant, the results were consistent with those seen in human monocyte-derived cells, thereby giving confidence in the *G. mellonella*–MAH infection model. A recent description of a method to generate multiple MAH transposon mutants found that 96% of the interrupted genes essential for *in vitro* growth had mutual orthologs in *M. tuberculosis* (Dragset et al. 2019). Additionally, the 3500 MAH transposon mutant library was assessed in a mouse model of infection, with 97% of identified virulence genes having mutual orthologs in *M. tuberculosis*. Thus, the method identified both virulence genes specific for MAH and shared with *M. tuberculosis*. Three MAH genes encoding a protein involved in nucleotide excision repair (UvrB), a probable major facilitator superfamily (MFS) transporter, and a hypothetical protein, were verified as virulence factors by wild-type versus mutant comparisons in the mouse model of infection. If there is a correlation between mutant attenuation in mice and *G. mellonella*, and the three mutants described above could form part of such studies, then the *G. mellonella*–MAH infection model would be useful to screen for virulence genes and identification of novel targets for therapy. Like other members of the MAC, it is also likely to be a good model for screening novel antimycobacterial agents, although this remains to be determined.

## *Galleria mellonella*-derived products for the treatment of tuberculosis

That both honeycomb and *M. tuberculosis* are waxy, and *G. mellonella* can dissolve honeycomb was the basis for Metchnikov’s suggestion that *G. mellonella* extracts could be used to treat tuberculosis (Metchnikov 1899). Metalnikov hypothesized that a *G. mellonella* lipase – he called cerase – was responsible for the decomposition of honeycomb and *M. tuberculosis* wax (Metalnikoff 1906). However, to this day, no pure cerase with such biological properties has been isolated, despite the efforts of many researchers (Feissinger 1910, Dublet 1921, Pertzoff 1928, Olivier 1947, Mankiewicz 1949, 1952, Kuzniecow and Wojciechowski 1950, Niemierko and Cepelewicz 1950, Annenkov et al. 2004). *Galleria mellonella* extracts prepared using acetone/glycerin/sodium hydroxide appear to have the best antimycobacterial activity *in vitro* and *in vivo*. However, the results are difficult to repeat because of the lack of experimental details, such as the larvae/adult moth quantity and/or the volumes of extraction reagents used. For example, Dublet (1921) merely refers to ‘a glycerin with added manganese extract’. Other researchers have prepared *G. mellonella* extracts that use organic solvents, such as acetone, alcohol, or ether, followed by evaporation under a partial vacuum that results in dried powder formulations (Kuzniecow and Wojciechowski 1950). Such an acetone/sodium hydroxide extract was active against BCG and H37Rv: activity lost upon storage in air or heating for 5 min at 100°C, suggesting the active ingredient(s) may be proteinaceous (Kuzniecow and Wojciechowski 1950). Another acetone/sodium hydroxide extract concentrated such that ‘1 c.c. corresponds to 1 gm of larval bodies’ was prepared by Olivier (1947) and had activity against ‘the tubercle bacillus of Arloing and Courmont’, which is most likely an *M. avium* strain (Griffith 1941). A common finding *in vitro* was growth inhibition with mycobacteria remaining intact but refractory to ZN-staining (Feissinger 1920, Dublet 1921, Pertzoff 1928, Olivier 1947, Mankiewicz 1952). In addition, *M. smegmatis* 607 preincubated in an ‘enzyme extract’ of *G. mellonella* larvae had decreased resistance to penicillin and sulphatiazole (Paszewski 1959). Notably, *G. mellonella* extracts have anti-*M. tuberculosis* activity in experimental animal models. For example, in guinea pigs, the ‘glycerin with added manganese *G. mellonella* extract’ prepared by Dublet (1921) prevented the development of tuberculosis, and prior treatment with an acetone extract of *G. mellonella* resulted in increased length of survival and a reduction in disease severity caused by H37Rv (Mankiewicz 1952). However, despite the activity of *G. mellonella*-derived ‘esterase preparations’ against H37Rv *in vitro*, no therapeutic effect in guinea pigs occurred (Annenkov et al. 2004). An added complication is that *G. mellonella* is known to produce a variety of AMPs (Mikulak et al. 2018), with 20 listed in the Antimicrobial Peptide Database (https://aps.unmc.edu/; date last accessed, 4 March 2023). To our knowledge, there has been no systematic evaluation of the AMP content of acetone/alcohol/glycerin/sodium hydroxide-derived *G. mellonella* extracts or assessment of individual *G. mellonella*-derived peptides against mycobacteria. A comprehensive chemical analysis of a 40% ethanol extract of *G. mellonella* larvae identified free amino acids, aromatic substances, fatty acids, nucleotides and nucleosides, peptide conjugates, trace metals, and a serine protease, but AMP content was not investigated (Spiridonov et al. 1992). Further research is required to determine whether *G. mellonella* AMPs singularly or combined, either in larvae or in *G. mellonella* extracts, contribute to any antimycobacterial activity found.

While there have been considerable efforts to identify a *G. mellonella* lipase with antimycobacterial activity, others have proposed that the bactericidal and/or bacteriostatic activity of *G. mellonella* extracts results from compounds produced by bacteria present in the larval gut flora (Paszewski and Jarosz 1976). Early culture studies found that *Streptococcus faecalis*, reclassified as *Enterococcus faecalis* in 1984 (Schleifer and Kilpper-Balz 1984), was the predominant species isolated from *G. mellonella* (Bucher and William 1967, Dudziak 1975). Abundant bacteria can persist throughout the insect’s metamorphosis (Johnston and Rolff 2015) and be transferred maternally to the eggs (Bucher 1963). Enterococci predominated in *G. mellonella* in a more recent 16S rRNA study, which analysed the skin, faeces (to represent the gastrointestinal tract), fat body and haemolymph of both ‘bait for fishing/pet food’ and ‘research grade’ larvae. In both types of larvae and all sites, one *Enterococcus* taxon dominated (relative abundance >50%), putatively identified as *Enterococcus gallinarum* or *Enterococcus saccharolyticus* (Allonsius et al. 2019). A new related species, identified by whole genome sequencing, with the proposed name of *Enterococcus innesii* sp. nov, was also recently isolated from *G. mellonella* (Gooch et al. 2021). *Enterococcus faecalis* presence has been suggested to be a natural component of nonspecific *G. mellonella* defence against bacterial infections, possibly through the production of lysosome and/or bacteriocins (Paszewski and Jarosz 1976). Additionally, ‘disabling’ the symbiotic bacterium *Enterococcus mundtii* in *G. mellonella* by antibiotic administration led to a microbiota dominated by pathogenic *Serratia* spp. and *Staphylococcus* spp., and early death (Johnston and Rolff 2015). Similarly, ingestion of an antibiotic cocktail of ampicillin, erythromycin, gentamicin, and kanamycin led to transcriptional activation of gallerimycin and metalloproteinase in the midgut, suggesting a role for the microflora in maintaining a healthy gut (Mukherjee et al. 2013). These results suggest that commensal and/or symbiotic bacteria can influence the outcome of infection; if the presence of enterococci or other bacteria can affect *G. mellonella*–mycobacteria interactions is unknown. Antimycobacterial substances are produced by bacteria isolated from *G. mellonella*; e.g. filtrates from 4/42 bacterial strains had activity against *M. smegmatis* 607 (Jozwik 1973). Filtrates from the most active strain, named 4-BIS and identified as a *Bacillus* sp., were subsequently found to be active against six *M. tuberculosis* strains, including H37Rv; no antimicrobial agent was identified. Similarly, a polypeptide-like antibiotic termed gallerin with modest activity against *M. smegmatis* 607 was isolated from *Bacillus subtilis* strain 26a (Paszewski and Jarosz 1976). Primycin is an antibiotic with activity against *Staphylococcus aureus* and mycobacteria derived from an ‘*Actinomycetes*’ strain, renamed as *Streptomyces primycini*, which was isolated originally from *G. mellonella*, and is a mixture of homologous nonpolyene polyketide molecules (Vályi-Nagy et al. 1954). However, mammalian toxicity and formulation issues from poor water solubility have prevented its use to treat systemic tuberculosis, but it was successfully used to treat urogenital tuberculosis (Kelenhegyi et al. 1956). Today primacin (a single component) is produced commercially for inclusion in topical products such as Ebrimycin gel to prevent bacterial skin infections, e.g. in burns treatment (Papp et al. 1990).

**Figure 7.**
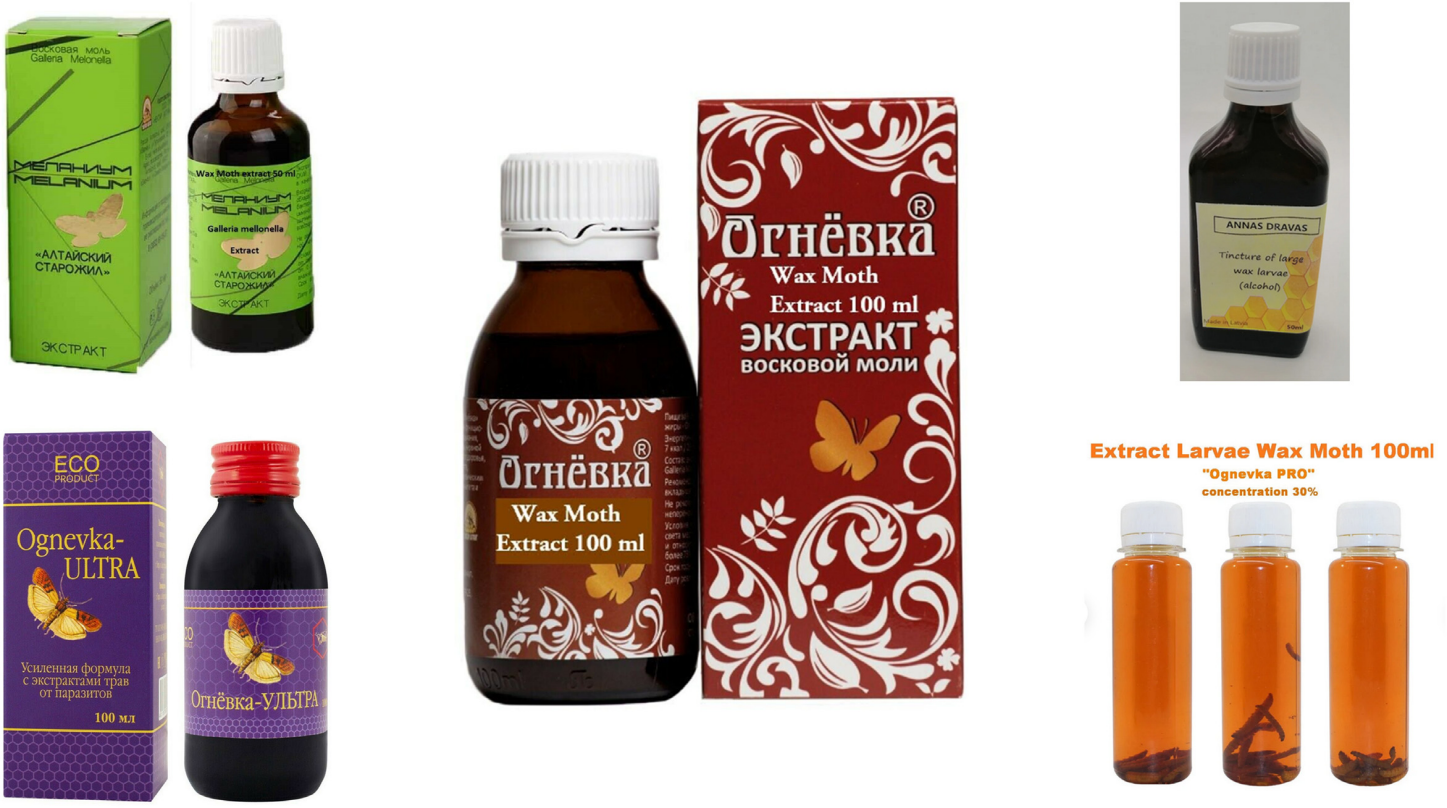
Screenshots of packaging of commercially available *G. mellonella* extracts. Except for Annas Dravas (top right-hand corner), produced in Latvia, all the others are of Russian origin. *Galleria mellonella* extracts are widely advertized as being suitable for the treatment of pulmonary disease and/or tuberculosis.

In summary, *G. mellonella* larval and adult moth extracts can decompose both honeycomb and the waxy lipids of *M. tuberculosis*. However, no definitive evidence that a lipase(s) in extracts is responsible for the anti-*M. tuberculosis* activity observed in some studies is available. The studies are hard to interpret because of the variety of extraction methods, lack of standardization and quality control of extracts, and the likelihood that the strains used in some studies are not *M. tuberculosis* as reported. It is also unclear whether the presence of commensal enterococci or other bacteria can influence the response of *G. mellonella* to mycobacteria. An added complication is that the microbiome of *G. mellonella* is affected by diet (Krams et al. 2015). Experimentally, the use of axenic (free of bacteria) *G. mellonella* (Waterhouse 1959) may elucidate a role for microflora in *G. mellonella*–mycobacteria interaction. Despite the many unknowns, in some parts of the world, noticeably Eastern Europe, *G. mellonella* extracts are available commercially and advertised for tuberculosis treatment (Fig. 7). We contend that such use is not evidence-based, there being a need for controlled randomized trials and, if there was a proven benefit, considerable work is still required to elucidate the underlying antimycobacterial mechanisms.

## *Burkholderia pseudomallei* and *Burkholderia mallei*

*Burkholderia pseudomallei* and *B. mallei* are Gram-negative intracellular bacteria, which cause aggressive disease in humans and animals (Schell et al. 2007). Research on *B. pseudomallei* and *B. mallei* is restricted to BSL-3 due to concerns over their use as biological weapons (Rotz et al. 2002). Genetically, *B. mallei* is a deletion clone of *B. pseudomallei*, with their genome differing by only 0.5% (Jeddeloh et al. 2003). *Burkholderia pseudomallei* is the causative agent of melioidosis, and a wide variety of clinical manifestations have been reported ranging from localized abscesses to acute pneumonia and septicaemia (Wiersinga et al. 2018). The incidence of melioidosis is most prevalent in Southeast Asia and Northern Australia, although cases are also widely reported in tropical and subtropical countries (Limmathurotsakul et al. 2016). Disease prognosis is generally poor, with ∼50% of individuals infected succumbing to the infection (Wiersinga et al. 2018). *Burkholderia mallei* causes a melioidosis-like disease known as glanders, primarily in horses, which typically in humans has a zoonotic origin with human–human transmission rarely recorded (Van Zandt et al. 2013).

*Galleria mellonella* use with *B. mallei* and *B. pseudomallei* was first described in 2008 (Schell et al. 2008). Larvae infected with 3–100 CFU of *B. mallei* reference strain ATCC 23344 had >90% larval mortality within 6 days. Infection with 10 CFU of *B. pseudomallei* strain KB96243 resulted in >80% death in 2 days with paralysis of infected larvae before death: a similar phenomenon occurs in hamsters. *Burkholderia mallei* and *B. pseudomallei* established a proliferative infection where >10^6^ CFU of bacteria were recovered from the larvae (Schell et al. 2008). *Galleria mellonella* can also discriminate virulence between *Burkholderia* species other than *B. mallei* and *B. pseudomallei*: there was a clear differentiation between comparisons of *B. pseudomallei, Burkholderia thailandensis*, and *Burkholderia oklahomensis* isolates (Schell et al. 2008, Wand et al. 2011). *Burkholderia thailandensis* and *B. oklahomensis* are genetically similar to *B. pseudomallei*, with *B. thailandensis* adopted as a popular surrogate for *B. pseudomallei* (Haraga et al. 2008). The virulence of *B. pseudomallei* (most virulent), *B. thailandensis*, and *B. oklahomensis* (least virulent) in *G. mellonella* mirrored that found in macrophage and mouse models of infection (Wand et al. 2011). *Galleria mellonella* was further demonstrated as a model to study *B. mallei* virulence through wild-type and isogenic mutant comparisons. Inactivation of key virulence genes: type II secretion system, type III secretion system, type IV secretion system (T4SS), and capsule all led to prolonged larval survival relative to wild-type, results comparable to those found in the hamster model (DeShazer et al. 1999, Ulrich and DeShazer 2004, Schell et al. 2007, 2008). Similarly, a double deletion mutant unable to express RelA and SpoT, which lacks (p)ppGpp-synthesizing enzymes, was defective in intracellular replication in mouse macrophages and was attenuated in acute and chronic mice, and *G. mellonella* infection models (Müller et al. 2012).

Following establishment, characterization, and standardization (Sprynski et al. 2014), researchers have utilized the *G. mellonella*–*B. pseudomallei*/*B. mallei* models to study mechanisms of disease. The previously undetermined role of trehalase (TreA), an enzyme of a pathway that converts trehalose into glucose, in *B. pseudomallei* virulence, was identified through comparative virulence studies in *G. mellonella* of wild-type and a Δ*treA* mutant. The attenuation of the Δ*treA* mutant in *G. mellonella* mirrored that found in mouse and macrophage infection models (Vanaporn et al. 2017).

*Galleria mellonella* use also helped to elucidate the roles of phospholipase C (Plc) enzymes during *B. pseudomallei* infection (Srinon et al. 2020). Plc enzymes are virulence factors involved in pathogenesis responsible for mediating/regulating tissue colonization, immune evasion, and induction of inflammation (Bandana et al. 2018). Three *B. pseudomallei* Plc enzymes are known; Plc1 and Plc2 have roles in nutrient acquisition and Plc3 in virulence. Single deletion mutants (Δ*plc1*, Δ*plc2*, and Δ*plc3*) and wild-type were equally virulent in *G. mellonella*. However, Δ*plc12* and Δ*plc123* mutants had significantly reduced virulence in *G. mellonella* relative to the wild-type, indicating a redundancy mechanism (Srinon et al. 2020).

The bacterial factor BimA is known to play a role in actin-based motility; it is required for movement within and between host cells (Jitprasutwit et al. 2016). In *B. pseudomallei*, BimA is regulated by the VirAG two-component system, as are Type VI secretory system-associated virulence factors (Burtnick and Brett 2013). The associated bacterial factor BimC and its role in virulence had not previously been characterized (Srinon et al. 2019). *In vitro* data indicated that BimC is vital for actin-based motility and for intracellular survival of *B. pseudomallei* in HeLa cells but not in macrophages. Furthermore, *bimC* deletion prevented plaque and multinucleated giant cell formation impairing its virulence. In *G. mellonella* infected with Δ*bimC*, larval survival improved significantly over the wild-type with reversion to near wild-type levels with complementation.

*Burkholderia pseudomallei* possesses a wide variety of mechanisms for intracellular survival, and a transcriptional study revealed novel genes upregulated during *in vitro* infection of macrophages. Most of these genes were located on chromosome 2 and associated with adaptation to and survival in intracellular environments (Ong et al. 2004). Two genes, *bpss1622* (a type III secretion system cluster 2 component) and *bpss2104* (a T4SS cluster 3 component) were selected for deletion as they had not been studied previously (Jitprasutwit et al. 2020). Infection of *G. mellonella* larvae with Δ*bpss1622* or Δ*bpss2104* resulted in attenuation, although the potential underlying mechanism(s) were not discussed (Jitprasutwit et al. 2020).

Serial passage of bacterial cultures is a regular practice amongst researchers to maintain an active culture or to harvest large numbers of bacteria. Prolonged passage is known to impact bacterial genotype or phenotype, resulting in alteration to virulence (Duangurai et al. 2020). Sequencing of *B. mallei* cultures serially passaged *in vitro* over 10 days identified the introduction of tandem repeat genome mutations (Romero et al. 2006). In addition, a comparison of *B. pseudomallei* DNA sequence generated from a 2004 stock with its daughter stock in 2018 revealed substantial changes to the genome (Wagley et al. 2019). To determine if *B. pseudomallei* virulence is affected by serial passage, *G. mellonella* larvae were infected with the 1st, 5th, and 28th passages of fresh clinical or reference *B. pseudomallei* (Duangurai et al. 2020). Larval survival significantly declined, indicating that *B. pseudomallei* virulence increased with serial passage. *In vitro* passage of *B. pseudomallei* enhanced properties associated with cellular invasion and intracellular survival. Changes in virulence were associated with host proinflammatory cytokine expression: verified by proteomic and transcriptomic analyses (Duangurai et al. 2020).

*Galleria mellonella* larvae were used to investigate the pharmacokinetics and efficacy of antibiotics used to treat *B. pseudomallei* (Thomas et al. 2013). Pharmacokinetic profiles of clinically relevant compounds, i.e. ceftazidime, CIP, doxycycline, and imipenem in *G. mellonella* haemolymph, were determined by well-diffusion assays; the conclusion was that the model approximates single dose antibiotic responses in humans. In addition, three novel fluoroquinolone compounds were evaluated for their ability to treat *G. mellonella* larvae infected with *B. pseudomallei*. Treatment was 2 h postinfection, and efficacy was measured 24 h posttreatment. All treatments substantially improved larval survival relative to no treatment controls, demonstrating the model’s usefulness in screening novel anti-*Burkholderia* agents (Thomas et al. 2013). Finally, two auranofin analogues active against *Burkholderia* clinical isolates *in vitro* were of low toxicity in *G. mellonella*, warranting further investigation for their therapeutic potential (Maydaniuk et al. 2021).

## Coxiella burnetii

*Coxiella burnetii* is a Gram-negative obligate intracellular bacterium that causes Q fever, a zoonotic disease with global distribution. The bacterium can infect many species, including arthropods, humans, and ruminants. In humans, Q fever presents as an acute self-limiting febrile to a chronic life-threatening disease, such as endocarditis. In farm animals, Q fever causes late abortion and reproductive disorders such as premature birth and dead or weak offspring (Maurin and Raoult 1999).

*Coxiella burnetii* phase I and II strains produce lipopolysaccharide, i.e. full length (smooth, complete with O-antigen sugars) or truncated (missing outer core and O-antigen), respectively. The transition from phase I to phase II occurs with *in vitro* passage and is associated with a permanent chromosomal deletion (Hoover et al. 2002). The Nine Mile II (NMII, phase II) RSA439 strain is widely used for research since it is usable at BSL-2, grown axenically, random transposon mutant libraries can be generated, and it has virulent properties in *in vitro* culture infection models (van Schaik et al. 2017). Its parent NMI (phase I) strain must be used at BSL-3. Whereas NMI is virulent in guinea pigs, nonhuman primates, and immunocompetent and SCID mice, NMII is virulent in SCID mice (van Schaik et al. 2017). In *G. mellonella* larvae, NMI and NMII were equally virulent: there was no significant difference in survival at any dose (Norville et al. 2014). Administration of doxycycline, used clinically to treat Q fever, 24 h postinfection with NMII, resulted in a significantly extended median time to larval death. The results suggested that the model is suitable for evaluating novel antibiotics to treat Q fever (Norville et al. 2014). In addition, the virulence of wild-type NMII and isogenic *dotA* and *dotB* transposon mutants were compared in *G. mellonella* larvae. The Dot/Icm T4SS is important for intracellular replication of *C. burnetii* (Beare et al. 2011), and *dotA* and *dotB* mutants are known to be replication-defective within epithelial cells (Martinez et al. 2016). At 216 h postchallenge, all wild-type larvae were dead; most of those infected with *dotA* or *dotB* mutants survived. In natural infection, macrophages are the prime target cells where the bacteria reside and replicate within lysosome-derived *Coxiella*-containing vacuoles (CCVs). The CCVs enlarge with bacterial replication and occupy much of the infected host cells. Norville et al. (2014) found, 48 h postinfection, that NMII cells were in large parasitophorous vacuoles within haemocytes where intracellular replication had occurred. They concluded that ‘*C. burnetii* induced killing was associated with bacterial persistence within haemocytes, which was at least partially dependent on the T4SS’.

A further study in 2014 involving *G. mellonella*, showed that OmpA, the first *Coxiella* invasin described, is required for virulence. *In vitro*, transposon mutations in *ompA* strongly decrease *Coxiella* internalization and replication within host cells (Martinez et al. 2014). In *G. mellonella* larvae, an NMII *ompA* transposon mutant was killed significantly faster than the wild-type. Also reported was that wild-type NMII induced large highly infected nodules of haemocytes frequently juxtaposed to severely infected and damaged larval organs, with individual bacteria visible at higher magnification being ‘reminiscent of what we had previously observed in cultured macrophages’. Infection with the *ompA* mutant resulted in fewer and smaller nodules. Thus, similar results were found in the *G. mellonella* infection model and *in vitro* (Martinez et al. 2014).

Most studies using the *G. mellonella*–*C. burnetii* infection model have compared the virulence of wild-type NMII and transposon mutants in T4SS-dependent effectors or associated proteins. Wild-type *C. burnetii* induces one large CCV within an epithelial cell. However, in host autophagy-deficient cells infected with wild-type NMII or host autophagy-replete cells infected with a *C. burnetii cig2* transposon mutant, infected epithelial cells contained multiple CCVs (Newton et al. 2014). The data suggested that Cig2, also called *Coxiella* vacuolar protein B (CvpB), an effector protein delivered into host cells by the T4SS, subverts the host autophagy machinery, and a single (homotypic) CCV results (Kohler et al. 2016). In *G. mellonella* haemocytes, a similar effect occurs in epithelial cells, i.e. homotypic and multiple CCVs are induced by wild-type and *cig2* mutants, respectively. In addition, compared to the wild-type, the time to death of *G. mellonella* larvae was significantly longer for two independent *cig2* mutants: reversed by a functional *cig2* gene in *trans*. Despite the effect on virulence, there was no significant difference in genome equivalents (a measure of bacterial load) between wild-type, *cig2*, or complemented mutants, indicating that intracellular replication does not require Cig2. The results suggested that Cig2 mediates host tolerance of infection. Martinez et al. (2016) also described the attenuation of NMII *cig2* mutants in *G. mellonella*. Experiments with epithelial cells established that Cig2 binds phosphatidylinositol 3-phosphate and phosphatidylserine on CCVs and endosomal membranes, which perturbs recruitment of phosphatidylinositol 5-kinase PIKfyve, which results in homotypic CCV formation (Martinez et al. 2016). Whether Cig2 mediates homotypic CCV formation in *G. mellonella* haemocytes by the same mechanism is unknown.

The effect of mutation of the NMII *CBU2072* (*eirA*) gene, which encodes the essential for intracellular replication A (EirA) protein, has also been investigated in *G. mellonella* (Kuba et al. 2020). As the name suggests, *eirA* (transposon) mutants have a severe intracellular growth effect and do not form CCVs (Newton et al. 2014). A total of four strains were investigated for virulence in *G. mellonella*: wild-type, *eirA*::Tn mutant, pFLAG-EirA, and a pFLAG-EirA24-165 (which has a truncated putative N-terminal signal sequence). Within 11 days all larvae infected with the wild-type and the pFLAG-EirA mutant had died, while those infected with the *eirA*::Tn and pFLAG-EirA24-165 mutants survived. Wild-type and pFLAG-EirA, but not *eirA*::Tn and pFLAG-EirA24-165 mutants, induced CCV formation, similar to results in epithelial and macrophage cell lines. Loss of EirA results in lack of translocation of T4SS-dependent effector proteins, suggesting a role in T4SS activity. Overall, the results suggest Eir is essential for intracellular replication in mammalian cells and *G. mellonella* and is dependent on the presence of the putative N-terminal peptide sequence identified.

**Figure 8.**
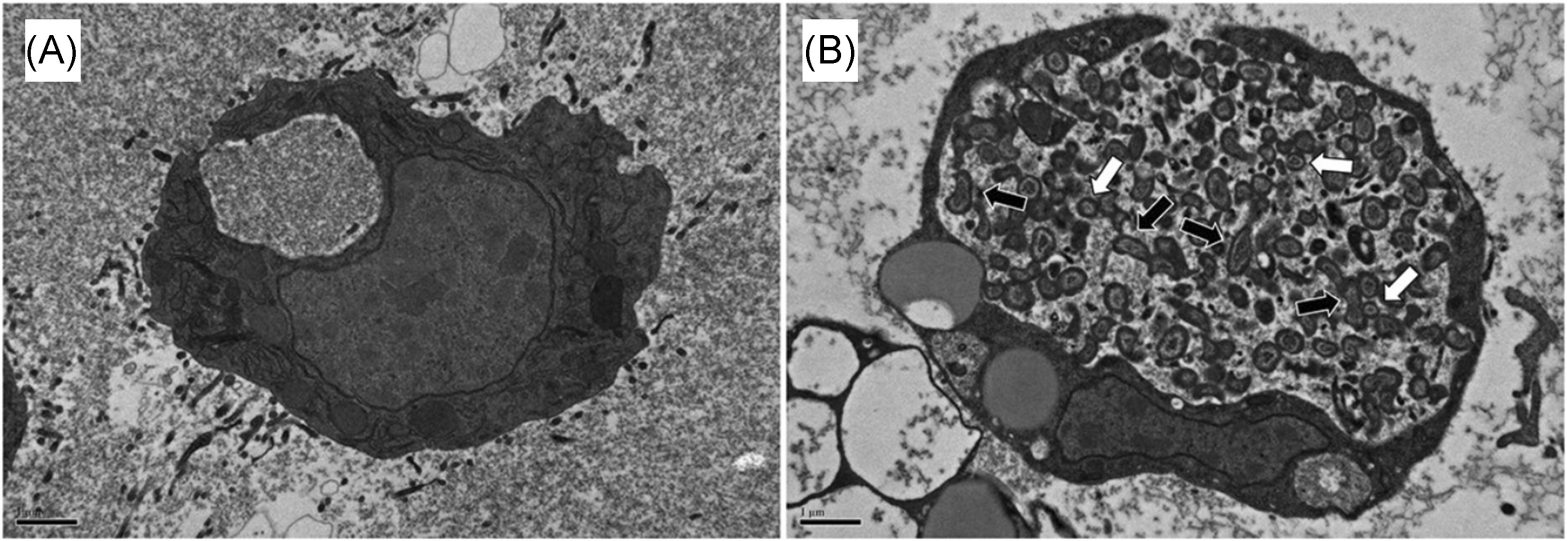
TEM of *G. mellonella* haemocytes 3 days postinfection with *C. burnetii*. (A) Uninfected controls with no visible bacteria. (B) Haemocytes from infected larvae, including a clearly visible CCV that fills the entire cell cytoplasm. Arrows indicate proposed LCVs (black) and SCVs (white) – see main text for further details. The data shown represent the analysis of 50 control and 50 infected haemocyte images. Scale bars = 1 μm. Reproduced with minor adaptation from Kovacs-Simon et al. (2020) with permission under CC-BY-4.0.

The transcriptome of NMII isolated from *G. mellonella* haemocytes was compared to the challenge inoculum grown in axenic culture, and 46 significantly upregulated genes were identified across all time points (Kovacs-Simon et al. 2020). The encoded proteins are predicted to be involved in translation, synthesis of biotin and lipopolysaccharide, scavenging of reactive oxygen species, and 30 hypothetical proteins, with some, e.g. those involved in biotin synthesis known to be important for *C. burnetii* virulence (Moses et al. 2017). The high expression of seven genes involved in translation was attributed to the faster growth rate in *G. mellonella* (mean generation time 5 h) compared to those grown axenically (mean generation time 10.6 h). In addition, 30 T4SS effectors were significantly upregulated in *G. mellonella*, of which 11 are upregulated in Buffalo Green Monkey (BGM) cells and mice (Kuley et al. 2015). Kovacs-Simon et al. (2020) also described the presence of both metabolically dormant nonreplicating small cell variants (SCVs), and metabolically active and replicating large cell variants (LCVs) in CCVs within haemocytes (Fig. 8A and B) and were morphologically similar to those found in *C. burnetii*-infected macrophages.

To survive in the host *C. burnetii* inhibits apoptosis, i.e. the removal of infected host cells, and the T4SS-dependent effector AnkG is a known bacterial antiapoptotic mediator (Klingenbeck et al. 2013). In *G. mellonella*, a Δ*ankG* mutant was significantly attenuated compared to the NMII wild-type. Compared to the wild-type, a *C. burnetii* mutant overexpressing FLAG-tagged AnkG showed reduced killing: no differences in infection or replication in infected haemocytes were found. These results are similar to those found in mammalian cells (Schafer et al. 2020). However, further analysis is required to determine if AnkG has antiapoptotic activity in *G. mellonella*. Similarly, CaeB is a known *C. burnetii* T4SS effector protein that inhibits apoptosis in mammalian cells by preventing endoplasmic reticulum stress-induced cell death (Friedrich et al. 2021). In *G. mellonella*, an NMII Δ*caeB* mutant is attenuated. Compared to the wild-type, there was no difference in Δ*caeB* infection rate. However, there was reduced CCV size and intracellular replication of the Δ*caeB* mutant in haemocytes. Again, whether the same antiapoptotic mechanism operated in *G. mellonella* and mammalian cells was unclear.

Finally, one study compared the virulence of ruminant (cow, goat, and sheep) isolates of *C. burnetii* to NMI (Selim et al. 2018). Isolates were classed as low, middle, or high virulence (which included NMI) despite no significant differences in LD50s. No relationship between virulence and the genomic group was possible because of a lack of genotypic data (Metters et al. 2019). The LD50 for NM1 was ∼3.5 logs lower than Norville et al. (2014) reported, which was attributed to possible differences in the immune status of the larvae in the two studies (Metters et al. 2019).

Overall, the above studies show that the *G. mellonella*–*C. burnetii* infection model is valuable for wild-type mutant comparisons and assessing antibiotic treatment, particularly in the whole animal context. The model has many features in common with *in vitro* and *in vivo* mammalian models. However, further work is required to determine whether the same bacterial and host pathogenic mechanisms operate in mammals and *G. mellonella*.

## Francisella tularensis

*Francisella tularensis* is the causative agent of tularemia transmitted by arthropods to humans and other vertebrates. There are three subspecies of *F. tularensis* but only *F. tularensis* subsp. *tularensis* (Type A) and *F. tularensis* subsp. *holarctica* (Type B) are definitively associated with human disease; it is unclear with *F. tularensis* subsp. *mediasiatica*. The genus also includes environmental members, considered opportunistic pathogens as they only infect immunocompromised individuals, i.e. *Francisella novicida, Francisella hispaniensis*, and *Francisella philomiragia*; the latter also implicated in bacterial infections in near-drowning victims (Sjöstedt 2007, Thelaus et al. 2018). Whether *F. novivida* is a subspecies of *F. tularensis* or a separate species is under debate (Kingry and Petersen 2014). Whole genomic sequencing and clinical and epidemiological data support their separation (Öhrman et al. 2021). *Francisella tularensis* subspecies are facultative intracellular bacteria, with designation as a potential biological weapon stimulating research. Tularemia usually presents with nonspecific symptoms of chills, fever, and headache. An ulcer may develop at the site of inoculation depending on the route of infection, and lack of treatment leads to enlargement of the draining lymph nodes, which may eventually suppurate (Sjöstedt 2007). Respiratory infection also occurs, which can be life-threatening, with a 50% case fatality rate if untreated.

Experiments in *G. mellonella* are limited to those looking at virulence and antimicrobial treatment. Injection into the haemocoel of *F. tularensis* subsp. *holartica* Live Vaccine Strain (LVS) resulted in dose and temperature-dependent killing, with increased larval survival at the lower temperature of 30°C. The use of antilipopolysaccharide antibodies demonstrated the association of LVS with *G. mellonella* haemocytes (Aperis et al. 2007). One study confirmed the effect of dose and temperature on *G. mellonella* larval survival but surprisingly found that *F. novicida, F. hispaniensis*, and *F. philomiragia* were significantly more virulent than *F. tularensis* subsp. *holarctica* and *F. tularensis* subsp. *tularensis*. The inverse relationship between the lethality for *G. mellonella* larvae and the ability to cause incapacitating disease in humans was attributed to the capacity of environmental species to utilize invertebrate hosts (Thelaus et al. 2018). Their *F. philomiragia* data contradicted an earlier study, which showed less virulence, requiring a 10-fold higher LD50 dose than the *F. tularensis* subspecies (Propst et al. 2016). However, dosages, methods, and strains differed, complicating any meaningful comparison between the two studies. *G. mellonella* has also been used to evaluate *F. novicida* mutants, demonstrating the importance for virulence of ubiquinone biosynthesis (Kazemzadeh et al. 2021) and the type VI secretion system and three of its effectors (Brodmann et al. 2021). Similarly, an *F. novicida qseC* mutant was attenuated in *G. mellonella*, showing that the cognate protein QseC (a two-component system sensor kinase) is a virulence factor (Dean and van Hoek 2015). Antimicrobial testing in *G. mellonella* has also been evaluated, including in the original description of a *G. mellonella–Francisella* infection model where treatment with CIP, levofloxacin, or streptomycin before or after infection decreased the tissue burden of *F. tularensis* in the haemocoel and prolonged survival (Aperis et al. 2007). Improved survival of *G. mellonella* larvae also occurred when azithromycin was given 2 h after *F. novicida* or LVS infection, compared to those treated with CIP (Ahmad et al. 2010). The polar nature of fosmidomycin limits its antibacterial activity as it results in poor penetration of cells, and it requires a transporter for uptake. A lipophilic prodrug version of acetylated fosmidomycin (called ‘compound 1’) showed GlpT transporter-independent killing of *F. novicida in vitro* and *G. mellonella* (McKenney et al. 2012). Finally, a screen of 420 FDA-approved drugs identified the polycyclic antidepressant maprotiline as having antivirulence activity against *F. novicida*. It prolonged the survival of *G. mellonella* infected with a lethal dose of bacteria when given four daily treatments of the drug (Dean and van Hoek 2015). The effect of maprotiline was more than expected based on antimicrobial activity alone. Thus the *G. mellonella*–*Francisella* infection model can be used to identify virulence genes and to screen for antibiotics with *in vivo* activity.

## Listeria monocytogenes

*Listeria monocytogenes* is a Gram-positive facultative anaerobic bacterium that, following ingestion of contaminated food, causes mild to severe gastroenteritis. However, in vulnerable individuals, e.g. children, the elderly, immunocompromised, and pregnant women, sepsis, meningitis: foetal infection and abortion can also occur (Radoshevich and Cossart 2018). The bacterium concerns food producers through its ability to grow at low temperatures, withstand environmental stress, and its high fatality rate. Survival in the environment and the host depends on a complex array of virulence factors that gives the bacterium the unique ability to cross the intestinal, blood–brain and fetoplacental barriers and is a paradigm in cell, functional, and infection biology (Radoshevich and Cossart 2018).

## *Galleria mellonella* as an infection model for *L. monocytogenes*

The first evaluations of *G. mellonella* as an infection model for *L. monocytogenes* were reported in 2010 (Fedhila et al. 2010, Joyce and Gahan 2010, Mukherjee et al. 2010). No *G. mellonella* mortality was observed in oral (10^8^ CFU) force-fed *L. monocytogenes* 2C strain – a bioluminescent derivative of the widely used clinical isolate 10423S, and was hypothesized to be due to the lack of the specific InlA invasin receptor E-cadherin in *G. mellonella*, which is essential to cross the intestinal barrier in humans. However, a 10423S *inlA* mutant was as virulent as the wild-type after injection directly into the haemocoel (Fedhila et al. 2010). Compared to the lack of larval death associated with oral feeding (Fedhila et al. 2010), there were differences in lethality between pathogenic *L. monocytogenes* strain EGDe and nonpathogenic *Listeria grayi, Listeria innocua, Listeria seeligeri*, and *Listeria welshimeri*, after injection dorsolaterally into the haemocoel of last-instar larvae at a dose of 10^6^ CFU (septic infection model) (Mukherjee et al. 2010). In addition, the *G. mellonella*–*L. monocytogenes* model was tested for its ability to distinguish between the virulence of different serotypes using known highly virulent or attenuated strains in mice. The most pathogenic in *G. mellonella* was a serotype 4b strain, which had a significantly higher killing rate than a serotype 1/2a strain (EDGe). Serotypes 4a, 4c, and 4d were of lower virulence, with results mirroring those found in the mouse infection model (Mukherjee et al. 2010). The authors also found that the septic infection model could, through comparison of wild-type and EGDe isogenic mutants in known virulence genes, e.g. in the pathogenicity island *vgc*, be used for comparative virulence assessment and therefore useful for identification of novel *L. monocytogenes* virulence factors. A similar study, also using EGDe and isogenic mutants, confirmed the utility of the *G. mellonella*–*L. monocytogenes* septic infection model, including identifying the *hly* gene that encodes the haemolysin listeriolysin O (LLO), which is required for escape from the primary vacuole into the cytoplasm of infected human cells (Radoshevich and Cossart 2018), as is necessary for growth and toxicity in *G. mellonella* (Joyce and Gahan 2010). A nonpathogenic *Lactococcus lactis* expressing LLO also killed *G. mellonella* larvae corroborating the results (Joyce and Gahan 2010). In addition, in isolated haemocytes, compared to the wild-type, no intracellular bacteria had actin ‘tails’ or ‘clouds’, which, in humans, are required for motility and cell-to-cell spreading. Unsurprisingly, an *actA* mutant did not have actin tails or clouds in *G. mellonella* (Mukherjee et al. 2013). EGDe was also chromosomally tagged with the *Photorhabdus luminescens* bioluminescence operon (*luxABCDE*) under the control of nine different promoters, allowing examination of the dynamics of virulence gene expression. For example, expression of *hly* was barely detectable in broth but induced within 30 min in *G. mellonella* with a peak at 7 h. Broadly, the results of Joyce and Gahan (2010) and Mukherjee et al. (2010) with the EDGe strain were similar, indicating the reproducibility of the model. However, one difference between the studies was *L. innocua* strain Clip 11262 was lethal for *G. mellonella* at comparatively high (Mukherjee et al. 2010) or low (Joyce and Gahan 2010) infectious doses. This difference in lethality for the same strain was attributed to the use of laboratory-reared (Mukherjee et al. 2010) versus out-sourced (Joyce and Gahan 2010) *G. mellonella*, the latter reared in the presence of antibiotics, and was suggested are ‘virtually unchallenged by microbes until exposure in our experiments’ (Joyce and Gahan 2010). Supporting the suggestion is that preexposure of *G. mellonella* to lipopolysaccharide decreases susceptibility to *L. monocytogenes* infection (Mukherjee et al. 2010). Rakic Martinez et al. (2017) further evaluated the *G. mellonella*–*L. monocytogenes* septic infection model and found that nonpathogenic *Listeria* spp. were significantly less virulent than *L. monocytogenes*. A dose of 10^6^ CFU enabled the comparative virulence of wild-type and attenuated in-frame deletion mutants (including *hly*) in a 14203S strain background to be determined. Thus, virulence studies of *L. monocytogenes* versus nonpathogenic *Listeria* spp. and validation with wild-type and isogenic mutants in different genetic backgrounds have established the utility of the *G. mellonella*–*L. monocytogenes* septic infection model.

## Virulence of different *L. monocytogenes* populations in *G. mellonella*

The *G. mellonella*–*L. monocytogenes* septic model has been used by others to assess the virulence potential of isolates of *L. monocytogenes* derived from different sources and belonging to different clonal complexes (CCs) (Kuenne et al. 2013, Rakic Martinez et al. 2017, Cardenas-Alvarez et al. 2019, Kong et al. 2019). Cardenas-Alvarez et al. (2019) analysed 34 mainly human and cow isolates from CC1, CC6 (lineage I), and CC7, CC9, CC14, CC37, and CC204 (lineage II) classified by the clinical outcome, i.e. bacteraemia, central nervous system, or maternal–neonatal infections, predominantly of serotype 1/2a or 4b, in the septic *G. mellonella*–*L. monocytogenes* infection model and identified CC14 maternal–neonatal-associated strains as being hypervirulent. The authors hypothesized that hypervirulence of CC14-maternal–neonatal-associated isolates was due to their ability to initially cause a low-level infection enabling evasion from the early *G. mellonella* immune response, which allowed for subsequent dissemination. Another study, using three different doses, found *L. monocytogenes* food isolates had significantly lower virulence for *G. mellonella* than paired clinical isolates, and noninvasive isolates from gastroenteritis outbreaks were significantly lower in virulence than those of the same serotype from outbreaks with invasive symptoms (Rakic Martinez et al. 2017). A study of 16 *L. monocytogenes* strains of different serotypes, 11 of which had their whole genome sequenced, were compared in the *G. mellonella* septic infection model with those from serotypes 1/2c, 3a, 3b, 3c, and 4b, and classified as virulent (Kuenne et al. 2013). Two serotype 1/2a human listeriosis outbreak strains were only of low virulence, which the authors suggested was a limitation of *G. mellonella* in predicting human infection. Most were lethal for *G. mellonella* larvae, but toxicity for Hela cells was variable. Genome analysis did not establish a possible explanation for the phenotypes observed (Kuenne et al. 2013). A particularly challenging study was to compare baseline transcriptomes of *L. monocytogenes* isolates of different lineages grown to exponential phase in brain heart infusion (BHI) broth with their virulence potential in the *G. mellonella*–*L. monocytogenes* septic infection model (Kong et al. 2019). Initially, 91 phylogenetically divergent isolates were assessed in *G. mellonella* using a standard dose of 10^6^ CFU, the readout being the time in days to kill 50% of larvae (LT50), with the majority (*n* = 65) killing in 2–3 days. There was no correlation between lineage or epidemiological background (epidemic, faecal, or food-related samples) and LT50. Based on LT50s and Maury’s classification (Maury et al. 2016), which categorizes *L. monocytogenes* CCs as hypovirulent, intermediate or unknown, and hypervirulent, the transcriptomes of 33 diverse but representative isolates grown in BHI broth to exponential phase at 37°C (considered representative of that *in vivo*) were determined (Camejo et al. 2009). The data identified genes whose expression level correlated with lineage, Maury’s classification, and LT50. A total of 56 differentially expressed genes, 39 and 17 significantly up or downregulated, respectively, were identified. None had been described previously as virulence-related. Predicted functions or identities of encoded proteins indicated involvement in carbohydrate transport and metabolism, defence mechanisms, energy production, lipid metabolism, translation and posttranslational modification, and a permease and a histidine triad protein. A total of 26 genes encoded predicted proteins that were ‘similar to unknown proteins’ or had ‘no similarity or information not available’. The authors acknowledged that although there were correlations between gene expression and LT50, no molecular marker predicted virulence. However, the study did identify genes potentially essential for *L. monocytogenes* virulence, at least in *G. mellonella*, which could be prioritized for wild-type versus isogenic mutant comparisons.

## Use of the *G. mellonella–L. monocytogenes* infection model to identify novel virulence genes

As indicated above, the *G. mellonella*-*L. monocytogenes* septic infection model was validated by comparison of wild-type *L. monocytogenes* with isogenic mutants known to be attenuated in other infection models (Joyce and Gahan 2010, Mukherjee et al. 2010, Rakic Martinez et al. 2017). The two largest studies were those of Mukherjee et al. (2010) and Rakic Martinez et al. (2017), who compared the virulence of wild-type with nine (EDGe background) and 11 (10403S background) isogenic mutants, respectively. There was overlap between some of the genes investigated, e.g. *hly, prfA* (encodes a master regulator of many virulence genes), and *actA* (encodes a protein that mediates host actin polymerization and intracellular motility), and attenuation occurred in both backgrounds. Therefore, it is unsurprising that many researchers have used the wild-type versus isogenic mutant approach to determine whether a specific gene of interest has a role in virulence. All of the following have been assigned as virulence factors in *G. mellonella* based on the approach: LmOf1875 ATP-binding cassette (ABC) transporter (Liu et al. 2022), penicillin binding protein 4 involved in copper tolerance, CadA4, a protein involved in cadmium tolerance (Parsons et al. 2017), UspA a universal stress protein (Seifart Gomes et al. 2011), SmpB (small protein B) involved in translation (Mraheil et al. 2017), and a putative P-type ATPase named FrvA (Fur regulated virulence factor A) (McLaughlin et al. 2012).

## Use of the *G. mellonella–L. monocytogenes* infection model to identify probiotic strains and plant products to prevent *L. monocytogenes* infection

The *G. mellonella*–*L. monocytogenes* infection model has also been used to evaluate the probiotic potential of non-*Listeria* strains and plant-derived products to prevent infection. Three lactic acid bacteria (LAB) strains, *Lactobacillus pentosus* B281 and *Lactobacillus plantarum* B282 (from table olive fermentations) and *Lactobacillus rhamnosus* GG (from the human intestinal tract) were evaluated for their probiotic potential to prevent *L. monocytogenes* B129 (a food isolate) infection in *G. mellonella* (Grounta et al. 2016). Specifically, injected live or heat-killed LAB and cell-free supernatants were used. The biggest decrease in B129 CFUs (a 1.8 log reduction) occurred after killed B281 or B282 were injected 6 h before the challenge. Cultured filter supernatant treatment derived from any of the LAB strains did not increase the survival of B129. A similar study investigated the ability of prior injection of either *L. rhamnosus* GG or *Clostridium butyricum* Miyairi 588 to protect against challenge with a lethal dose of an *L. monocytogenes* serotype 4b gastrointestinal isolate (Scalfaro et al. 2017). Pretreatment with either probiotic strain increased larval survival after the challenge with *L. monocytogenes*. Increased haemocyte density, a measure of the immune response, was found in the pretreated larva. *In vitro*, agar spot tests found that the probiotic strains had inhibitory activity for *L. monocytogenes* growth. The authors suggested that the probiotic activity *in vivo* was due to immune enhancement, *in vitro* inhibition to antibacterial activity by an unknown mechanism, and the use of *in vitro* and *in vivo* approaches provides complementary information.

The ability of plant products such as eugenol (4-allyl-2-methoxyphenol), an ingredient of clove oil that has Generally Recognized as Safe (GRAS) status, alone or in combination with LAB (*Lactobacillus* or *Bifidobacterium* probiotic strains) to protect *G. mellonella* against infection against three (two of which were serotype 4b) *L. monocytogenes* strains has been assessed (Upadhyay et al. 2016). The protocol involved the injection of subinhibitory concentrations of eugenol plus or minus *L. monocytogenes* culture supernatants immediately before the *L. monocytogenes* challenge. Challenge was with a dose that resulted in 100% mortality by 5 days. Eugenol and supernatants derived from all LAB strains significantly enhanced larval survival compared to controls. Supernatant from a *Bifidobacterium bifidum* strain had the most protective effect of any LAB and was enhanced when combined with eugenol. A similar study design used by the same group showed that subinhibitory concentrations of the phytochemicals trans-cinnamaldehyde, present in cinnamon bark extracts, and the antimicrobials carvacrol and thymol present in oregano oil, all significantly enhanced *G. mellonella* survival when challenged with lethal doses of three *L. monocytogenes* strains (Upadhyay et al. 2016). The most effective treatments were 0.1% concentrations of carvacrol and trans-cinnamaldehyde. A different approach involved assessing comparative virulence in *G. mellonella* of an *L. monocytogenes* serotype 1/2a blood isolate grown in conventional growth media with or without a subinhibitory concentration of a steam-distilled essential oil of *Cannabis sativa* L. (cannabis oil) (Marini et al. 2018). Larval survival was highest in groups challenged with the isolate grown with cannabis oil. The results were consistent with other antivirulence traits associated with subinhibitory cannabis oil in the same study, e.g. lack of mobility, reduced biofilm formation, and invasion of CaCo-2 epithelial cells. Thus, all three studies show that *G. mellonella* has a potential role in screening for probiotics that prevent *L. monocytogenes* infection for inclusion in further animal and/or human trials. Ultimately, the validation of the approach will come from recognition by screening in *G. mellonella* and successful use in well-controlled human trials.

## Growth medium effects on *L. monocytogenes* virulence in *G. mellonella*

Investigating the effect of different relevant environmental growth conditions on the ability of *L. monocytogenes* to infect *G. mellonella* has also been studied (Schrama et al. 2013, Rakic Martinez et al. 2020). Cheese processing can expose *L. monocytogenes* to low pH due to organic acids, such as L-lactic acid and/or acetic acid, and high salt. Investigation of four *L. monocytogenes* isolates incubated in a cheese-based medium with acid (pH 5.5) or sodium chloride (3.5% w/v) habituation, found that both stresses resulted in two isolates having significantly decreased and equivalent lethality for *G. mellonella*, compared to nonhabituated controls (Schrama et al. 2013). In contrast, habituation in L-lactic acid or acetic acid before injection into *G. mellonella*, increased the virulence (as adjudged by decreased LT50s) of two foodborne outbreak isolates compared to nonhabituated controls (Li et al. 2021). The discrepancy in results was attributed to isolate variation and suggested the need for further *L. monocytogenes* to be studied to determine the extent of the phenomenon. Major USA *L. monocytogenes* foodborne outbreaks were associated with cantaloupes and Granny Smith apples prompting an investigation as to whether growth on these is associated with increased virulence in *G. mellonella* compared to conventional BHI-broth grown cells (Rakic Martinez et al. 2020). A total of 14 *L. monocytogenes* strains of three major disease-causing serotypes, 1/2a, 1/2b, and 4b, were evaluated. Compared to BHI-grown cells, there was a significant increase in mortality of *G. mellonella* when infected with *L. monocytogenes* grown on cantaloupe and apple slices, which was dose and strain-dependent, and independent of incubation temperature (10°C and 25°C). The increased virulence was more pronounced with cantaloupe-grown *L. monocytogenes*. Some, but not all, strains grown on apples killed *G. mellonella* in a shorter time, but surviving larvae lived longer than BHI controls. The mechanisms underlying the increased virulence remained obscure. However, bacterial numbers were lower in the cantaloupe compared to the BHI-grown group, suggesting that it was not the number of cells *per se* that caused the increase in virulence but changes in gene expression. Transcriptomic data describing an upregulation in virulence genes, including an upregulation of the *prfA* regulon, in cells grown on cantaloupes compared to those in BHI, supports the assertion (Kang et al. 2019). The results of all the above studies show that growth on different foods can affect the phenotype of *L. monocytogenes*, with the implication that increased virulence may have the consequence of a reduction in infective dose and/or increased severity of disease (Rakic Martinez et al. 2020).

## Immune response of *G. mellonella* to *L. monocytogenes*

Understanding the immune response of *G. mellonella* to non-native pathogens, such as *L. monocytogenes*, is important to determine their relevance to human infection, drive uptake contributing to replacement/reduction of animals used in infection studies, understand strengths and limitations of *G. mellonella*-infection models, and for a greater understanding of insect immunity. The original papers describing the *G. mellonella– L. monocytogenes* septic infection model also analysed the *G. mellonella* immune response to *L. monocytogenes* (Joyce and Gahan 2010, Mukherjee et al. 2010). Both found that *L. monocytogenes* infection was accompanied by melanization, resulting from induction of the PPO cascade, and a significant decrease in haemocyte numbers, which did not occur after infection with attenuated *L. monocytogenes*. Haemocyte decrease was hypothesized to be linked to *G. mellonella* driven by LLO activity (Joyce and Gahan 2010). *Galleria mellonella* haemolymph is known to contain inducible anti-*Listeria* activity (Mukherjee et al. 2010), in part, through AMP action. Such defence mechanisms are presumably overwhelmed when there are fewer haemocytes, as occurs in the presence of high numbers of bacteria. Both groups found *L. monocytogenes* induced expression of *G. mellonella* genes important for immunity, including those encoding the AMPs galiomycin, gallerimycin, gloverin, and inducible metalloproteinase inhibitor (IMPI), lysozyme, transferrin, peptidoglycan recognition protein B, and PPO subunit 2. Induction of genes encoding galiomycin, gallerimycin, IMPI, lysozyme, and another AMP produced by *G. mellonella*, cecropin B, were also induced by heat-killed *L. monocytogenes*, and synthesized cecropin B was subsequently shown to have a growth inhibitory effect *in vitro* (Mukherjee et al. 2011). However, wild-type versus isogenic mutant studies suggested that some *L. monocytogenes* genes, e.g. *mprF* and members of the *dlt* operon, involved in the synthesis and addition of D-alanine to cell wall-associated lipotechoic acids, respectively, counteract AMP activity (Mukherjee et al. 2011).

An early observation was that *L. monocytogenes* infection of *G. mellonella* larvae delays their transition to pupae and a role for histone acetyltransferases (HATs) and histone deacetylases (HDACs), which promote and repress gene expression, respectively, in this phenomenon has been described (Mukherjee et al. 2012). Experiments with HAT and HADC inhibitors and RT-qPCR analyses of genes involved in this epigenetic process found that *L. monocytogenes* infection resulted in a persistent skewed HAT/HDAC balance. The authors concluded that *L. monocytogenes* ‘manipulate’ histone acetylation in *G. mellonella* to suppress immune responses that, through epigenetic control, collaterally impact development. The word ‘manipulate’ suggests an active process, but the mechanisms underlying this phenomenon were unknown. It was proposed LLO had a role since it can inhibit histone acylation and phosphorylation in epithelial cells (Alvarez-Dominguez et al. 1997). A later study found that *L. monocytogenes* infection of *G. mellonella* resulted in the induction of genes mediating binding or processing of either juvenile hormone or ecdysone, principal mediators of metamorphosis, and was consistent with the developmental delay observed (Mukherjee et al. 2013). Alteration in HAT/HDAC balance was also associated with wounding suggesting that the phenomenon is non-specific, supported by the finding that a lethal challenge with *L. monocytogenes* and preactivation of larvae with lipopolysaccharide (not present in *L. monocytogenes*) induces similar immune responses (Mukherjee et al. 2010).

microRNAs (miRNAs) have also been implicated in regulating the *G. mellonella* immune response to *L. monocytogenes* infection (Mannala et al. 2017). The use of an insect-specific miRNA microarray with >2000 probes identified 90 miRNAs (39 upregulated and 51 downregulated) in response to infection with EGDe. A subset of four miRNAs (miR-133, miR-954, miR-998, and miR-3000), which had mammalian homologs or had known or predicted functions, were validated by RT-qPCR. Except for miR-3000, exposure to nonpathogenic *L. innocua* did not alter miRNA expression, indicating ‘a concept of virulence-dependent miRNA regulation during host-microbe interactions’. Subsequently, putative target genes were predicted, their gene expression determined, and mRNA–miRNA duplex structures modelled. There was an excellent inverse agreement between *L. monocytogenes* induced up- and downregulation of cognate miRNAs. Downregulation of miR-300 was associated with upregulation of *Optineurin* (involved in autophagy) and *MAP-kinase* (involved in PPO induction), and downregulation of miR-198 was associated with upregulation of *Optineurin*, and *Spätzle*, a receptor of the Toll pathway (involved in AMP induction). A role for autophagy in restricting *L. monocytogenes* infection in *G. mellonella* is indicated by prior treatment with rapamycin, which induces autophagy, resulting in a reduction in *L. monocytogenes* within haemocytes (Mukherjee et al. 2013). Upregulation of miR-954 and miR-3000 was associated with the downregulation of *CYP4G1* and *CYP6B4* genes, respectively, which encode xenobiotics involved in toxin and drug metabolism. While the identity of the target genes of the differentially expressed miRNAs needs to be confirmed, the results indicate a role for miRNA regulation of the *G. mellonella* immune system in response to infection by pathogenic bacteria.

**Figure 9.**
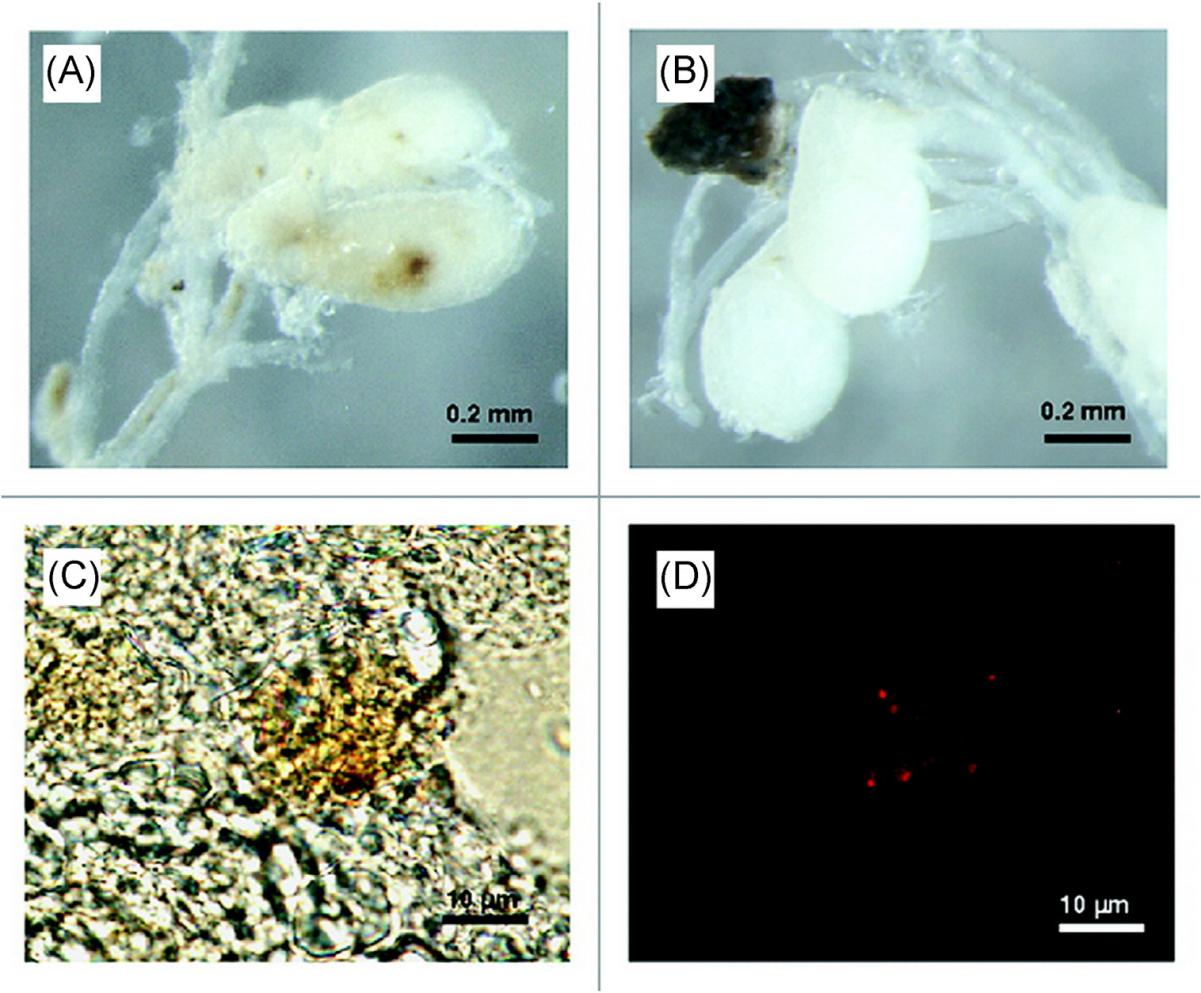
*Galleria mellonella* brain infected with *L. monocytogenes*. (A) Dissection of *G. mellonella* larvae infected with *L. monocytogenes* reveals the formation of dark spots in the brain. (B) Treatment with diclofenac prevents the formation of these spots. (C) Localized melanized regions form in the *G. mellonella* brain following infection with *L. monocytogenes*. (D) Persistence of labelled *L. monocytogenes* (red fluorescence) in melanized regions. Reproduced from Mukherjee et al. (2013) with permission.

Dorsolateral injection into the haemocoel of last-instar larvae results in septic shock and alterations in the brain of *G. mellonella*, mimicking aspects of meningitis in humans (Mukherjee et al. 2013) (Fig. 9A–D). Brains were dissected 6 days postinfection with a dose of 10^6^ CFU of EDGe, and there was clear evidence of ‘melanized nodules similar to those observed in the integument’. *Listeria monocytogenes* expressing a red fluorescent protein established bacterial survival within nodules. Administration of diclofenac, a nonsteroidal inflammatory drug, inhibited nodule formation. The results indicated an immune response to *L. monocytogenes* occurs in the brain. Transcriptome analysis identified the induction of genes, including AMPs and heat shock proteins, and those involved in cell proliferation and neuronal repair, oxidative stress responses, and phagocytosis. Administration of diclofenac or rapamycin at the onset of infection improved *G. mellonella* survival, showing that the induced immune response restricts *L. monocytogenes* pathogenesis (Mukherjee et al. 2013). This work was described ‘a highly significant advance’ in the use of *G. mellonella* larvae since it ‘opens the possibility of examining neural disease and repair mechanisms’, and ‘for rapidly evaluating the efficacy of novel drugs designed to counter a variety of neural diseases or malfunction’ (Browne and Kavanagh 2013). Arguably, its potential has not been realized in these areas, but *G. mellonella* use will likely increase.

Thus the *G. mellonella* immune response to *L. monocytogenes* is complex, but has parallels with humans, hence its wide use in dissecting both host and bacterial factors that contribute to the infectious process.

## Future prospects

This review has highlighted the extensive work undertaken using *G. mellonella*–intracellular bacteria pathogen infection models (summarized in Table 1), particularly for evaluating antimicrobial activity *in vivo* and comparative virulence. We envisage that the use of such models will increase, particularly given the recent decision of the FDA to no longer require testing of new drugs in animal models, although it is predicted that this will take years to have an impact (Wadman 2023). Concerning novel antimicrobials: little use of *G. mellonella* to assess toxicity has been made, possibly reflecting the lack of novel agents currently in development. Given the excellent correlation between the toxicity of chemicals in *G. mellonella* and mammals (Allegra et al. 2018, Piatek et al. 2021), there is also likely to be a substantial increase in this area.

**Table 1.** Summary of the activities undertaken that involve *G. mellonella* (GM) and the intracellular bacterial pathogens included in this review. See main text for further details.

Activity		*Burkholderia* spp.	*C. burnetii*	*Francisella* spp.	*Listeria* spp.	*Mycobacterium* spp.
Comparative virulence	Between different isolates and/or species	+	+	+	+	+
	Wild-type versus mutant	+	+	+	+	+
Antimicrobials	Efficacy testing	+	+	+	+	+
	Toxicity testing^1^	+	–	–	–	+
Probiotics	Efficacy testing^2^	−	–	–	+	–
Antimicrobial source	Derived from GM^3^	–	–	–	–	+
	Derived from GM bacterial flora^3^	–	–	–	–	+
Response to infection^4^	Bacterial	+	+	+	+	+
	GM	+	+	+	+	+

1 Where there was a specific aim to test for toxicity of an antimicrobial against the target bacterium.

2 Where there was a specific aim to test a probiotic to prevent infection against the target bacterium.

3 When used to test against the target bacterium.

4 Includes proteomic and/or gene expression and/or histopathological and/or microscopic analyses.

Overcoming current hurdles will also increase the use of *G. mellonella*–intracellular bacteria pathogen infection models. The availability of *G. mellonella* genomes and annotations are constantly improving with a new RefSeq genome recently deposited (December 2022) in GenBank (accession number: GCA_026898425.1). Genome availability will be invaluable in driving the development of reagents, e.g. antibodies and aptamers, and the design of molecular probes to detect and quantify informative immune markers, especially cytokines and chemokines, a current bottleneck. However, progress is being made, e.g. in the recent description of intracellular interferon-gamma detection in larval haemocytes (Wrońska et al. 2022). Detection was based on the cross-reactivity of rabbit monoclonals recognizing human interferon gamma with *G. mellonella* interferon gamma. Detailed protocols described the quantification and visualization of intracellular cytokine production using flow cytometry or fluorescence microscopy. Cross-reactivity between antibodies recognizing interleukin-1-alpha, tumour necrosis factor-alpha, and heat-shock proteins and their counterparts in *G. mellonella*, has also been reported (Wittwer et al. 1999, Wrońska and Boguś 2020). The availability of further reagents for the detection and/or assay of immune markers, such as cytokines and chemokines, perhaps through a *G. mellonella* community initiative in collaboration with industry, will also facilitate the uptake of *G. mellonella–*intracellular bacteria infection models.

Another major hurdle is the lack of a *G. mellonella* genetic toolkit to construct *G. mellonella* mutants or reporter strains. Methods are under development, such as those using PiggyBac transposase-mediated transgenesis to generate a strain expressing EGFP and DsRed markers, and CRISPR-Cas9 engineering to construct an EGFP mutant (Pearce et al. 2021), and RNAi has been used to knockout the *G. mellonella* IMPI gene (Grizanova et al. 2021). This situation contrasts with other invertebrate hosts, such as the genetically tractable *C. elegans* and *D. melanogaster*, used to investigate all the intracellular pathogens that are the subject of this review. While an in-depth discussion of *G. mellonella, C. elegans*, and *D. melanogaster* interactions with intracellular pathogens is beyond the scope of this review, the comparative advantages and disadvantages of the three invertebrates in infectious disease research have been discussed (Glavis-Bloom et al. 2012). An example of the power of a host mutant approach is it was found, through both RNAi inhibition and a *C. elegans* mutant, that the p38 mitogen-activated protein kinase (MAPK) is important for protection against *M. marinum* infection (Galbadage et al. 2016). Similar experiments with *D. melanogaster PGRP-SA^seml^* and *Dif^1^* mutants, which had significantly increased mortality compared to control flies after infection with *M. abscessus*, demonstrated a role for the Toll pathway in host defence against mycobacteria (Oh et al. 2013). Thus, the ability to make and availability of mutants of nonmammalian hosts, such as *G. mellonella*, will drive uptake. We also envisage that there will be an increase in studies screening bacterial transposon mutant libraries in *G. mellonella* to identify bacterial genes required for survival in the host. Transposon mutant libraries are available for all of the intracellular bacteria reviewed here, but, to our knowledge, none have been screened in *G. mellonella*. Particularly informative would be comparisons of a transposon library in different hosts, as was done with *M. marinum* and hosts of protozoan and vertebrate origin (Weerdenburg et al. 2015), since this allows for the identification of genes required for survival in single and multiple hosts.

Immune priming is another area anticipated to be a subject of increasing research interest. This phenomenon is where prior exposure to a nonlethal dose of a pathogen, pathogen-derived material, or stress renders immunity to a lethal dose a short time later (Cooper and Eleftherianos 2017, Sheehan et al. 2020). Protection may be homologous or heterologous, i.e. against the same or different pathogens, respectively. The mechanisms are currently obscure but may be mediated by increases in haemocytes and AMPs (Sheehan et al. 2020). Heterologous and homologous examples with *G. mellonella* and intracellular pathogens include lipopolysaccharide protecting against *L. monocytogenes* (Mukherjee et al. 2010) and low-dose BCG against subsequent lethal BCG challenge (Asai et al. 2021). In the latter case, only priming with BCG but not other bacteria afforded protection, raising the possibility that *G. mellonella* can be used to predict vaccine efficacy in humans and comparison of the protective efficacy of new BCG and/or whole-cell vaccines such as MIP and *Mycobacterium vaccae*, currently being evaluated in man (reviewed in Cho et al. 2021), with results in *G. mellonella* will establish any correlation. Experimentally, novel antimicrobials should also be tested for immune priming in *G. mellonella* to prevent inactive compounds from being incorrectly classified as active *in vivo* (Sheehan et al. 2020).

*Galleria mellonella* also has a pedigree as a model for studying how exposure to infection can influence natural selection, transgenerational immune priming (TgIP), i.e. the transfer of a protective effect from the parent to its offspring, and the role of epigenetics in response to infection (reviewed in Vilcinskas 2016). To our knowledge, no one has infected *G. mellonella* with the intracellular pathogens that are the subject of this review to select future generations of pathogen-resistant insects or to investigate TgIP. However, natural selection experiments have been reported with other bacteria such as *Bacillus thuringiensis* (Mukherjee et al. 2017) and TgIP, e.g. *Escherichia coli, Micrococcus luteus, Serratia entomophila*, and *Pseudomonas entomophila* (Freitak et al. 2014). Experiments with *E. coli* suggest TgIP is mediated by the maternal transfer of bacteria or bacterial fragments to the developing eggs (Freitak et al. 2014). There is evidence that epigenetic mechanisms, including DNA methylation, histone acetylation and deacetylation, and miRNAs, are involved in TgIP responses to infection (Vilcinskas 2016), particularly paternal TgIP (Vilcinskas 2021), although other possibilities have been suggested (Tetreau et al. 2019, Lanz-Mendoza and Contreras-Garduño 2022). Such epigenetic mechanisms are known to be involved in the *G. mellonella* response to infection with *L. monocytogenes*: detailed in the ‘Immune response of *G. mellonella* to *L. monocytogenes*’ section above, as they are in the human response to infection to that pathogen (reviewed in Bierne and Hamon 2020). Epigenetic mammalian host responses have also been described after infection of cell lines and/or animals with many intracellular bacterial pathogens (reviewed in Fol et al. 2020), including, relevant to this review, *B. pseudomallei* (Cizmeci D et al. 2016), and *M. tuberculosis* (reviewed in Fatima et al. 2022), where there is extensive interest because of the potential for developing new and diagnostic biomarkers and therapeutics (Sui et al. 2022). A recent study indicated that miRNAs have a role in the epigenetic reprogramming of innate immunity in *G. mellonella* larvae such that pathogenic and commensal *E. coli* could be distinguished (Mukherjee et al. 2020). It is likely that a similar degree of specificity of *G. mellonella* epigenetic response to individual intracellular bacteria and/or strains will occur, although this remains to be determined. The use of *G. mellonella* infection models to understand the role of epigenetics in natural selection, TgIP, and host response has significant advantages, including similarity to human responses and investigations of multiple generations in a short time (Vilcinskas 2016). Given that the subject area is of considerable current interest, we envisage that the advantages of using *G. mellonella* will drive uptake and lead to an increased understanding of host epigenetics in response to intracellular bacterial infection.

Other areas where we foresee an increase in understanding of *G. mellonella–*intracellular bacteria interactions are likely to arise because of the application of existing techniques or technological advances. Examples include the areas of metabolomics (Tounta et al. 2021), recently used to investigate metabolic changes in the fat body in response to immune priming (Wu et al. 2022), and imaging. Examples of imaging include the relatively simple time-lapse systems for monitoring *G. mellonella* responses to infection (Kay et al. 2019) to more sophisticated techniques such as Fourier-Transform Infrared spectroscopy (Zdybicka-Barabas et al. 2019, Réjasse et al. 2022). In addition, label-free multimodal imaging methods may be particularly informative. These include fluorescent lifetime imaging (FLIM), coherent anti-Stokes Raman scattering (CARS), two-photon excited fluorescence (TPEF), and second harmonic generation (SHG), all used in conjunction with histological images to analyse *G. mellonella* infection-associated tissue damage (Quansah et al. 2022). The methods were also applicable to bacteria growing as a biofilm, a mode of growth that may be important for *M. tuberculosis* survival *in vivo*, including caseous necrosis and cavity formation in lung tissue (Esteban and García-Coca 2018). A research priority in the area is to define the physiological state of *M. tuberculosis* in these biofilm-like ‘clumps’ and ‘clusters’ (Bacon et al. 2022), which are also seen in the *G. mellonella* model (Asai et al. 2022). Thus, determining whether the *G. mellonella*–*M. tuberculosis* infection model can be used to investigate any role of mycobacterial biofilms in pathogenesis is worthy of future research. In addition, computed tomography, magnetic resonance imaging, and positron emission tomography were persuasive high throughput techniques to monitor infection, inflammation, and response to antibiotic treatment of *Manduca sexta* tobacco hornworm (Windfelder et al. 2022) and may apply to *G. mellonella–*intracellular bacteria infection models. The imaging technique spatial transcriptomics, i.e. assigning cell types identified by mRNA readouts to their locations in histological sections (Rao et al. 2021), can also potentially transform our understanding of *G. mellonella–*intracellular bacteria interactions, a fully annotated genome will facilitate this. Using such techniques may answer fundamental questions such as those first posed in the 1920s as to whether some of the *G. mellonella* immune responses to intracellular bacteria are pathogen-specific or nonspecific.

## Concluding remarks

There was a golden era of *G. mellonella* infection model research with mycobacteria in the early-mid 20th century, as exemplified by the seminal papers of Metalnikov, and translationally some success, e.g. it indirectly led to the development of primamycin. Following a relative waning in the amount of research, there has been a resurgence in *G. mellonella*-infection models in the last 15–20 years. As indicated by the examples of intracellular bacterial pathogens discussed above, *G. mellonella*-infection models are valuable in many areas, including probiotic prophylaxis, assessment of plant products to prevent disease and for assessing *in vivo* efficacy of antibiotics and identifying virulence factors. However, researchers should recognize the strengths and weaknesses of *G. mellonella* infection models with their pathogen of interest, which will ultimately determine use. For example, the data suggest that the *G. mellonella–L. monocytogenes* septic infection model does not help investigate adherence mechanisms of the bacterium but does assist novel virulence gene discovery. Therefore, it is unsurprising that the approach of parallel use of *G. mellonella* with other infection models for a particular intracellular bacterial pathogen is a constant theme. Nonetheless, as indicated in the future prospects section, we predict that with the change in regulations for the translation of new compounds to clinical trials such that animal data is no longer mandatory, a new golden era of *G. mellonella*–intracellular bacterial pathogen research is upon us.
